# Non-invasive blood pressure monitoring using wearables for cardiovascular risk assessment: a systematic review

**DOI:** 10.1007/s00404-025-08301-2

**Published:** 2026-01-16

**Authors:** Michelle Zwahlen, Elena Pavicic, Kerstin Khattab, Valerija Krbanjevic, Julia Endrass, Petra Stute

**Affiliations:** 1 Faculty of Medicine, University of Bern, Bern, Switzerland; 2 Department of Obstetrics and Gynaecology, University Hospital of Bern, Bern, Switzerland; 3 Graduate School for Health Sciences, University of Bern, Bern, Switzerland; 4 Cardiance Clinic, Pfäffikon, Switzerland

**Keywords:** Cardiovascular disease, Wearable devices, Health monitoring, Blood pressure, Hypertension, Cardiovascular risk

## Abstract

**Purpose:**

Cardiovascular diseases are the leading causes of mortality in women worldwide, with hypertension being a major risk factor. While traditional blood pressure monitoring techniques rely on cuff-based measurements, wearable devices offer a promising alternative for continuous and non-invasive blood pressure tracking. This systematic review investigates the extent to which wearable blood pressure measurements can serve as surrogates for traditional sensors to be implemented in risk assessment tools in predicting cardiovascular risk in women.

**Methods:**

A systematic search was conducted in databases including MEDLINE, Embase, Cochrane Library, Web of Science, Scopus and ClinicalTrials.gov. Studies published between 2010 and 2024 were included. Exclusion criteria were case reports or animal studies. Study selection was performed based on PRISMA guidelines. Data extraction focused on wearable devices, measurement approach, validation against gold-standard BP methods, and their predictive utility for cardiovascular outcomes.

**Results:**

The systematic literature search revealed 14′863 results after removal of duplicates, of which 245 were selected for extraction. Most included studies used photoplethysmography, with pulse transit time and pulse wave velocity as core parameters, some using machine learning. Accuracy was moderate to high for diastolic blood pressure, but systolic blood pressure showed greater variability. Cardiovascular risk stratification showed promising results, though external validation was rare.

**Conclusion:**

Wearable blood pressure monitoring technologies are maturing, with growing potential in preventive cardiovascular medicine in women. However, clinical implementation is limited by varying accuracy, need for calibration, and the lack of standardization. Further validation and longitudinal studies are needed to establish their role in cardiovascular risk prediction.

**Supplementary Information:**

The online version contains supplementary material available at 10.1007/s00404-025-08301-2.

## Introduction

The World Health Organization (WHO) states that cardiovascular diseases (CVD) are the leading cause of death in men and women worldwide [1]. 30% of deaths in women are caused by cardiovascular diseases. This affects women of all ages, both fertile and during/after menopause. High blood pressure during pregnancy is a key risk factor for complications like pre-eclampsia and increases the risk of long-term hypertension, therefore, early detection and treatment are crucial. Due to hormonal changes during menopause the risk of developing CVD grows significantly, resulting in premature and preventable deaths [2].

Nevertheless, women are heavily underrepresented in studies on cardiovascular health and are often misdiagnosed as there is still the widespread misconception that CVD in women are less common than in men and females often report ‘atypical’ symptoms which are not recognised as signs of CVD. Therefore, primary prevention and early detection play a critical role [2].

The prevalence of hypertension, a major risk factor for CVD, is increasing globally [3]. Gold standards for BP estimation are cuff-based sphygmomanometers, providing point-in-time readings, but do not enable continuous monitoring, or intra-arterial catheters, which are invasive and therefore not applicable in ambulatory use [4]. To come up with the increasing demand for non-invasive, wearable and continuous BP monitoring wearable technologies have been developed.

The European Society of Hypertension (EHS) has emphasised the importance of core issues such as accuracy, performance, and implementation to fully leverage the potential of cuffless BP devices [5].

**Fig. 1:**
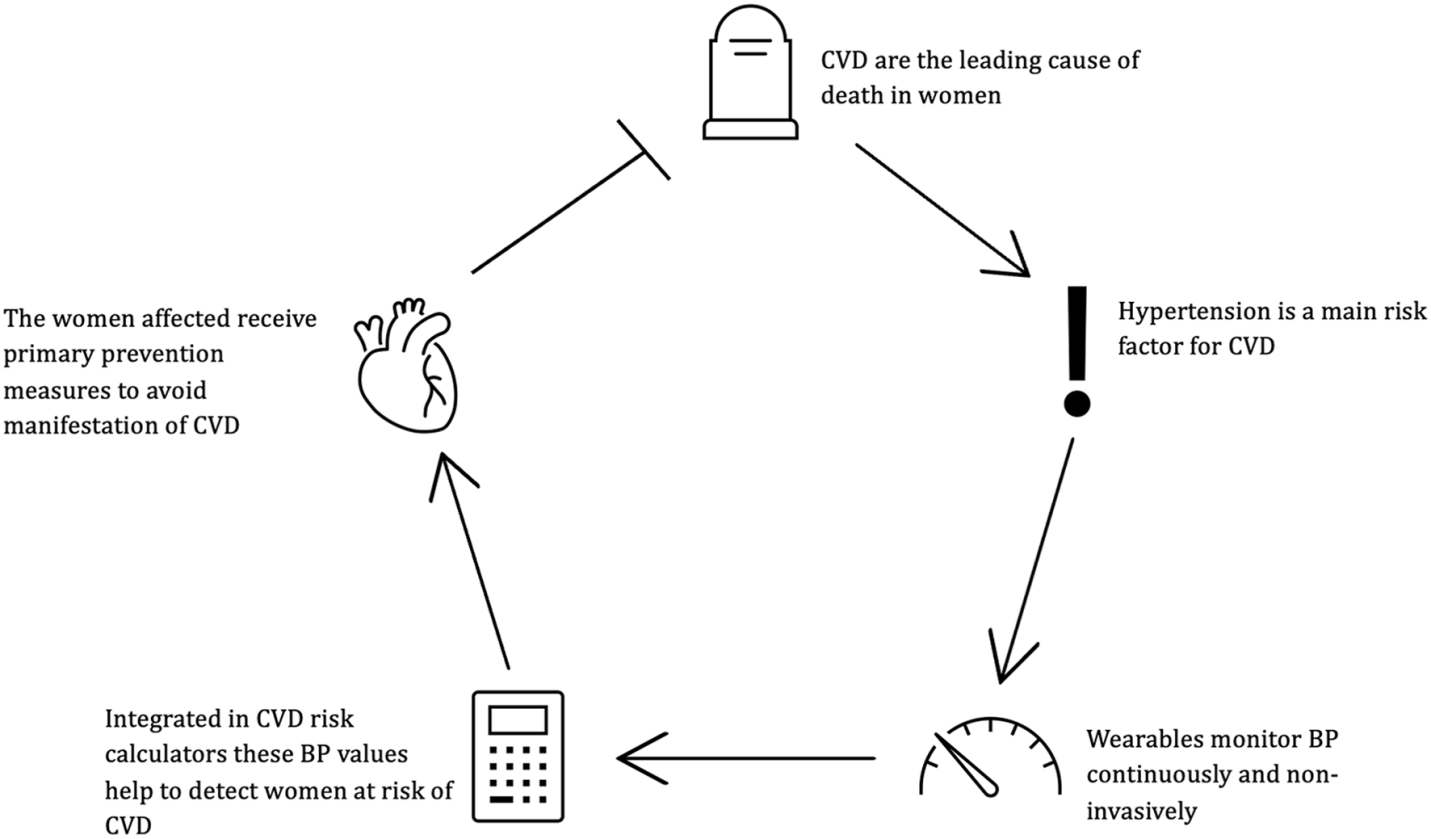
Conceptual pathway linking wearable blood pressure measurement to cardiovascular risk prediction

To reduce cardiovascular mortality, individuals at increased risk must first be identified. Hypertension represents a key modifiable risk factor and may, in principle, be detected using wearable blood pressure monitors, provided that these devices deliver valid and reliable measurements. Robust validation is therefore essential. Only with sufficiently accurate blood pressure data can wearable-derived measurements be meaningfully integrated into cardiovascular risk prediction models. This, in turn, may enable the identification of women at increased cardiovascular risk and facilitate timely preventive interventions (Fig. 1).

This systematic review aims to evaluate the validity of wearable BP measurements compared to conventional cuff-based methods. In addition, it investigates whether wearable-derived BP data can be considered a reliable surrogate for established clinical parameters used in traditional CVD risk prediction models.

## Methods

### Search strategy and eligibility criteria

A comprehensive literature search was conducted using MEDLINE All (Ovid), Embase (Ovid), Cochrane Library (Wiley), Web of Science Core Collection (Clarivate), such as the Science Citation Index Expanded (SCIE) & Emerging Sources Citation Index (ESCI), Scopus (Elsevier, and ClinicalTrials.gov (National Library of Medicine). The search strategy was based on a combination of controlled vocabulary (e.g., MeSH, Emtree) and free text terms, covering cardiovascular disease, risk assessment, blood pressure measurement, and wearable technology. The last search was conducted on May 1st 2024.

Due to the large amount of retrieved literature, the screening and data extraction stages were very time-consuming and required extensive collaborative effort. Therefore, we no further search update has been performed. The resulting time gap reflects the methodological thoroughness of the review and is in accordance with the Cochrane Handbook [6]. The remaining time has been used for the screening, data extraction and development of the manuscript. The full search strategies are available in the supplementary materials (Supplementary file 2).

The initial search strategy included an investigation of wearables for both the assessment of blood pressure and lipid status in cardiovascular risk calculators. Based on the amount of search results and the diversity of these topics, however, secondarily the topics were split into two separate papers as it is advised by the Cochrane Handbook [7]. The reported outcomes and risk of bias assessments represent the blood pressure-related studies solely. The screening process is demonstrated in the PRISMA-flowchart (Supplementary file 1).

Although this review aimed to address blood pressure estimation and cardiovascular risk prediction in women, studies including both sexes were retained, as sex-disaggregated analyses were rarely available in the existing literature.

To investigate the potential of BP monitoring in all its depth, we investigated both the technical aspects by looking at the validity of different BP sensors as well as the clinical application through CVD risk calculators. Only when combining valid BP sensors and accurate CVD risk calculators, early detection of cardiovascular risk and, therefore, preventive measures are possible.

### Inclusion & exclusion criteria

Inclusion criteria:

- Studies assessing wearable devices for blood pressure measurement.
- Studies evaluating the predictive value of wearable BP data for cardiovascular risk assessment.
- Original research, preprints, and systematic reviews published in English from 2010 to May 2024

Exclusion criteria:

- Animal studies.
- Studies in languages other than English.
- Case studies and non-peer-reviewed articles.

### Screening and data extraction

Title and abstract screening were conducted in Covidence [8] by independent reviewers, following a dual control principle. Full-text screening was performed, based on the mentioned eligibility criteria. Data was extracted by two independent reviewers. Device type, measurement method, validation against traditional BP measures, and predictive utility for cardiovascular risk assessment were noted in an Excel sheet (Supplementary file 3). As correlation coefficients and mean absolute error were variables stated in most papers, these values were used for comparability. Given the extreme heterogeneity of wearable blood pressure technologies, validation protocols, reference standards, outcome definitions and study populations, a quantitative synthesis was limited and a quantitative meta-analysis was not considered methodologically appropriate. Therefore, structured narrative synthesis was used to address topics where comparison or studies or mixing of studies seemed inappropriate, following the guidance from the Cochrane Handbook [9].

### Quality assessment

Study quality was assessed using appropriate risk of bias tools, including the Cochrane Risk of Bias tool for randomised trials^9^ and the Newcastle–Ottawa Scale for observational studies [10].

## Results

This review is part of a broader research project based on a comprehensive systematic search on cardiovascular biomarkers. A companion paper focusing on lipid-based biomarkers and their predictive value for cardiovascular outcomes has been submitted separately [11]. The last literature search was conducted on May 1st, 2024, retrieving a total of 22,903 records. After the removal of duplicate entries, the titles and abstracts of the remaining 14,863 studies were screened for eligibility. In total, 1,291 articles were selected for full-text screening, of which 252 publications were deemed eligible for this review. During data extraction, 7 of these papers, however, were found to be duplicates, therefore, 245 publications were included in this review eventually (Supplementary file 3 + 4).

Only 27 papers differentiated between female and male subjects in their results section and none of them reported sex-disaggregated accuracy metrics (Supplementary file 3).

In the first part of the results section, we will investigate the different types of BP devices and their validity, while we discuss their implementation in machine learning and risk stratification tools in the second part and the challenges sensors and machine learning algorithms are facing in the third part of this chapter.

### Risk of bias assessment

Risk of bias assessment revealed substantial heterogeneity across the methodology of sensor design and assessment and outcome measures of the included 245 studies. Generally, bias was high in prototype proposition papers due to a lack of necessary information. Both a selection and verification bias were common in sensor validation studies due to the inclusion of predefined normo- and hypertensive subjects or the inclusion of healthy and young participants only.

By omitting information about sex-disaggregated accuracy a systematic bias was introduced.

These issues were considered in the interpretation of the findings issued below, weighing more robust papers with greater weight than those with a higher risk of bias. A detailed overview of individual risk of bias ratings can be found in Supplementary file 4.

### Validation of wearable BP devices

Wearable BP monitoring devices rely on a wide range of sensor technologies, each with distinct mechanisms and limitations.

#### Photoplethysmography (PPG)

PPG sensors were discussed in 119 papers, making them the most common sensor type. Changes in blood flow affect the amount of light reaching the optoelectric sensor, altering the electrical signal output [12–14]. For BP estimation, parameters obtained from PPG data are:

- Pulse transit time (PTT), the time interval required for a pulse wave to propagate from a central to a distant measuring point [4, 15, 16]
- Pulse arrival time (PAT), the time interval between the onset of the electrical activity of the heart (the R-wave in the ECG) and the arrival of the pulse wave at the distal site[13, 17–20]
- Pulse wave velocity (PWV), the speed at which the pulse wave propagates, estimated by dividing PTT or PAT between two sensors by the distance between them[13, 17, 21]
- Pulse wave morphology for pulse wave analysis (PWA), using one-single sensor only[13, 22].

BP is calculated combining either PTT or PAT with ECG signals, analysing changes in PWV or investigating morphologic features of the pulse wave [23, 24].

Due to broad differences in sensor placement, data processing, study settings and measured outcomes, the variability in accuracy is large and the comparability limited. The mean absolute error (MAE), obtained by 21 papers, showed a range of 0.2-23 mmHg for SBP and 0.3–7.7 mmHg for DBP [25–27]. Due to the big differences in study designs and settings a direct comparison between these results is inappropriate, yet a trend towards better accuracy in the estimation of DBP than SBP is visible across the papers.

Correlation coefficients mentioned in 14 papers ranged between 0.55 and 0.98 for SBP and between 0.55 and 0.97 for DBP meaning moderate to high correlation between estimated and real BP values [28–30]. The paper reporting the highest Pearsons correlation coefficient for DBP, however, had a high risk of bias due to intransparency in their methodology [30].

#### Fiber Bragg Grating (FBG) Sensors

FBG sensors were researched in 4 articles. They rely on the reflection of a specific wavelength of light, which changes proportionally to the mechanical strain e.g. through blood volume changes applied to the fibre [31].

Only Katsuragawa et al. and Kumar et al. offered quantitative results, demonstrating a high correlation of > 0.9 for SBP in 3 subjects and an accuracy of 90% for SBP and 94% for DBP in 35 participants, respectively [32, 33].

Even though the sensitivity of the sensor seems promising in these small study cohorts and the sensor is both flexible and resistant to electromagnetic fields, the mentioned experiments had a high risk of bias as the sensors have not been tested in larger, more representative cohorts or in outpatient settings so far [31–33].

#### Ultrasound sensors

Ultrasound-based sensors were covered in 7 articles. They enable continuous blood pressure estimation by measuring real-time changes in arterial diameter corresponding with pulse waveforms, from which BP can be derived[12].

Analysis of their validity was only performed by Guo et al. and Wang et al. both in laboratory settings, demonstrating a mean difference of the mean arterial pressure (MAP) of -0.97 mmHg in 32 subjects and a mean estimation error of 1.6 mmHg for DBP at rest in 30 subjects, respectively [34, 35].

The advantage of ultrasound sensors is the high-resolution data of deep lying and, therefore, central arteries, inaccessible by other sensors, which may represent the actual BP more accurately than peripheral measurements [12].

#### Arterial applanation tonometry

Arterial applanation tonometry, covered by 12 articles, involves applying a controlled force over a superficial artery, measuring the resulting vessel wall deformation with a contact force sensor and analysing the thereby obtained waveforms [12, 23, 36].

These waveforms can either be analysed directly, often using machine learning algorithms, or by calculating PPG or PWV to estimate BP [4, 14, 37].

Quantitative results were only stated in two papers testing in different settings and for different parameters: Huang et al. demonstrated accurate results of 83% of SBP- and 87% of DBP-measurements within a 5 mmHg error margin, respectively [37]. Pielmus et al. combined arterial applanation tonometry with PPG and impedance photoplethysmography and fed the data into machine learning algorithms. The result was an absolute error of 4.5–4.6 mmHg [38].

#### Capacitive pressure sensor (CAP)

The CAP sensor, covered in 8 articles, measures the pulse wave by detecting changes in the electric state of the sensor introduced through strain [39, 40].

Two articles presented quantitative results: Abiri et al. demonstrated a correlation of 0.99 for SBP and 0.96 for DBP in comparison to gold standard intraarterial catheters in 15 patients on the intensive care unit and in the operating room[17]. Bijender et al. revealed that 98% of their measurements were within an error range of 10 mmHg in 130 participants in laboratory conditions [39].

#### Piezoelectric sensors

Piezoelectric sensors were represented in 12 papers. Under mechanical stress e.g. a pulse wave, they produce electric potentials, which can be translated into PTT or PWV to estimate BP or PWA is performed [41, 42].

Wang et al. developed and tested a piezoelectric system on 30 subjects, revealing a mean absolute error of 1.5 mmHg for SBP and 1.8 mmHg for DBP, respectively [43].

Sensors offer a low level of detection and fast response time, additionally they showed the potential of being self-powered [12, 42–44].

#### Accelerometric sensors

Accelerometric sensors were mentioned in 5 papers. They are used to record seismocardiographic signals (SCG), by capturing vibrations of the chest caused by cardiac activity [19]. Most commonly they were used in combination with other sensors (e.g. PPG sensors) to track and then remove motion artefacts [45]

#### Impedance cardiography and plethysmography (ICG/IPG)

Impedance cardiography (ICG) and impedance plethysmography (IPG) are mentioned in 16 papers. They monitor blood volume changes by measuring alteration in the ionic conduction i.e. the impedance, which is inversely related to blood volume. [46]. Based on PTT estimation, sometimes implementing machine learning algorithms or adding data from PPG-sensors BP is estimated [46–48].

Studies were performed in laboratory settings only and investigated different endpoints. The mean absolute error was stated in 3 investigations, ranging from 1.7 mmHg to 3.6 mmHg, however, only one of them differentiated between systolic and diastolic MAE reflecting a smaller error for DBP [47, 49, 50].

Advantages of this sensor type include reduced dependency on exact sensor placement, the need of only one sensor and the capability to monitor deeper arteries. Sensors are less susceptible to light and motion artefacts or looser skin contact [46].

### Machine learning and cardiovascular risk prediction

BP estimation represents only the initial step in evaluating an individual’s cardiovascular risk. Accurate acquisition and storage of vital parameters are essential, but by themselves do not improve clinical outcomes. To impact the patient’s health, the data needs to be interpreted correctly, and interventions must be made accordingly [51].

To analyse and contextualise the enormous amounts of data collected during continuous monitoring, machine learning algorithms are broadly implemented, ideally linking vital parameters to cardiovascular risk prediction tools. The appropriate machine learning tool needs to be selected depending on the sensor type and the demanded task as their strengths and weaknesses differ tremendously [47]. Machine learning algorithms were discussed in 34 articles (Supplementary file 5). The most used algorithms were different regression models with varying performance, even when applied to the same dataset [52]. Mujawar et al. attempted to explain the different capabilities of a few regression algorithms [53].

What most algorithms have in common is that they initially need to be trained on existing data, to teach them how to classify the input, before validating whether classifications are made accurately [54]. Combining different vital parameters and the inclusion of additional health record information can further increase accuracy [54, 55]. When trained correctly, many of these systems can work autonomically detecting changes in vital signs and therefore offer a big opportunity for early detection of cardiovascular diseases [56].

The main issues with machine learning algorithms are the intransparency of data analysis, as well as the dependency on accurate data input. Slapnicar et al. offer one of the few investigations comparing accuracy of in-hospital and outpatient estimation of BP. They showed that even when noisy data is removed (in their case 60% of outpatient data needed to be eliminated), accuracy of BP estimations was much lower in the outpatient setting [57]. This exemplifies why the development of accurate BP sensors is as crucial.

### Challenges

The integration of wearable-based BP monitoring systems into clinical practice presents several challenges. While these devices offer the advantage of continuous, non-invasive monitoring, multiple factors impact their accuracy and practicality.

#### Measurement inaccuracy and validation

A major concern with wearable BP devices is their measurement accuracy compared to the gold standard. Traditional BP measurement relies on oscillometric methods, whereas wearable devices use different approaches, the most frequent being PTT-based estimations using PPG sensors. By estimating BP through changes in values such as PTT or variation in pulse wave morphology, not BP itself is tracked, but rather changes in BP from the baseline inputs through calibration [5, 58, 59].

Several validation studies have highlighted the lack of accuracy in estimating SBP. Studies suggest that PPG-based methods often demonstrate higher accuracy for DBP but less reliability for SBP due to higher variability in signal processing [12].

Algorithm calibration and sometimes frequent re-calibration are necessary for most wearable devices, often requiring a cuff-based reference measurement, reducing the potential of a wearable sensor as a truly independent BP monitoring tool [4, 12, 20, 32, 42, 60].

#### Motion artefacts and environmental changes

As a key difference from point-in-time BP measurements, continuous monitoring poses new challenges like artefacts introduced through motion or changes in posture [5, 12, 19, 31, 35, 60–63].

While machine learning-based filtering techniques, changes in sensor architecture, or integration of accelerometric data into the algorithm could minimise these disturbances, as demonstrated in a small cohort by Phan et al. [45] motion-induced errors remain a key limitation [64, 65].

Optoelectric sensors are disturbed by ambient light exposure and interindividual variations in skin pigmentation [18, 58]. These variations make accurate BP measurements across diverse populations difficult.

Sensitivity to electrical or magnetic fields poses a further source of artefacts in piezoelectric sensors [43].

#### Skin contact and wearable fit

Many sensor types are susceptible to changes in device placement and depend on continuous application pressure to omit measuring errors [12, 14, 17, 36]. Depending on the sensor size, an unnoticed displacement of a few millimetres can result in measuring errors or signal loss [36, 66].

Sweat or dehydration resulting in alteration of ionic conduction, on the other hand, impacts sensor accuracy in impedance plethysmography sensors [20, 67].

#### Ethical challenges

Wearable BP devices collect and store sensitive health data on cloud-based platforms, raising ethical and security concerns in terms of risk of data breaches and unauthorized third-party access [12, 68].

To ensure patients’ control over their data, data encryption, use of firewalls and the assignment of personalised logins are implemented [16].

#### Comfort and user acceptance

Different sensor characteristics have the possibility to challenge user comfort and, therefore, acceptance. Both large and bulky sensors [12, 32] as well as systems with the need of multiple sensors in different locations can be uncomfortable for the user [14, 19]. Further systems that depend on continuous tight skin contact may cause skin irritation [12, 42, 68].

Many wearable BP devices require frequent charging or the change of batteries due to the high power consumption of optical sensors [18]. In the case of Florez et al., for example, the battery lifetime was listed as 12.5 h only, reducing the feasibility for 24/7 monitoring [69].

Further, especially for fragile or short-lived sensors of improving biodegradability is an important challenge, as many systems use environmentally toxic compounds [44].

Studies suggest that patients may abandon wearable BP monitoring due to inconvenience. Unlike traditional cuffs used intermittently, wearables that require continuous engagement, may lead to user fatigue and non-compliance over time [68].

## Discussion

This systematic review synthesises the current evidence on wearable-based BP monitoring technologies and their potential application in CVD risk stratification. This is a rapidly growing field with considerable promise, but also significant limitations that must be addressed before these devices can be broadly implemented in clinical practice.

### Heterogeneity and bias

Among the 245 included publications, a total of 92 studies focused primarily on the development of novel sensors or blood pressure (BP) estimation systems. As a result, a pronounced degree of methodological and technological heterogeneity was observed, which substantially limits direct comparability between studies. This heterogeneity is largely attributable to marked differences in sensor technologies, validation protocols, reference standards, signal processing approaches and outcome definitions.

In many cases, proposed sensor systems were evaluated only in preliminary feasibility studies involving small sample sizes and controlled laboratory conditions. While such early-stage investigations are essential for technological innovation, they inherently restrict the robustness of validation and limit external validity. Moreover, study populations most frequently consisted of young and healthy individuals, introducing a clear selection bias. Consequently, wearable BP systems were neither systematically tested in populations representative of clinical practice nor within the BP ranges of highest relevance for cardiovascular risk detection. Several studies explicitly reported larger estimation errors at hypertensive BP levels, likely due to the limited availability of corresponding reference measurements in these datasets [18, 70].

In addition, a verification bias was introduced in several validation studies through the predefined inclusion of normotensive and hypertensive subjects [63, 67, 70]. While this approach facilitates technical performance testing across a wider BP range, it may artificially inflate apparent accuracy and limits generalisability to unselected real-world populations.

Noticeably, in the entirety of the investigated publications, sex-disaggregated accuracy is missing completely. This issue does not mean that there is no difference between both sexes, but emphasises the lack of research and the underrepresentation of women in cardiovascular studies, resulting in the underdiagnosis of women [2].

### Accuracy and performance of wearable devices

With 119 references, PPG-based systems dominate the landscape. The variation in accuracy was large; however, several studies have demonstrated accurate approximation of BP compared to the gold standard [27, 71].

However, a consistent topic across the literature is that DBP is estimated with higher accuracy than SBP [15]. This discrepancy is likely due to the greater influence of motion artefacts, variability in signal morphology, larger spectrum and physiological factors (e.g., arterial stiffness) that disproportionately affect SBP [25]. Studies employing machine learning algorithms have improved estimation accuracy, especially when trained on multimodal sensor inputs (e.g., ECG + PPG), but these often require repetitive subject-specific calibration and lack generalisability [54]. Most sensors were tested in controlled settings only. Findings from these studies need to be handled with care, as the few authors who performed investigations in ambulatory settings as well agreed on the higher rates of error, increased difficulty introduced through more extensive noise due to movement and problems with recording phenomena like night-time dipping of BP [42, 57, 72]. Concerningly, some studies featuring hypertensive subjects found bigger errors in these exact subgroups than in normotensive subjects [15, 19, 21, 66].

Few studies adhered to international validation standards such as those proposed by the Institute of Electrical and Electronics Engineers (IEEE), which was designed specifically for cuffless BP sensors [36], or by the Association for the Advancement of Medical Instrumentation (AAMI), the European Society of Hypertension (ESH), or ISO 81060–2, which were created for the validation of cuff-based measurements. The absence of standardised validation and the extreme heterogeneity in study designs not only limits comparability between studies but also undermines regulatory progress. In a statement by the European Society of Hypertension Working Group, the use of the standards for cuff-based BP sensors was reported as misleading in the evaluation of sensors requiring to be calibrated. The reason being, that these devices ‘track’ BP based on the calibration rather than measuring it [5].

### From blood pressure to cardiovascular risk prediction

Beyond measurement accuracy, a core aim of this review was to assess the clinical applicability of wearable-derived BP values for CVD risk assessment. While many studies focused solely on validating BP measurements, a smaller subset explored predictive modelling for CVD. Farman et al., for example, used combinations of wearable data and electronic health record data to estimate the risk of cardiovascular disease with an accuracy of 98.5% [73].

Studies applying neural networks or random forest classifiers reported high accuracy in predicting hypertension or CVD risk categories [54]. However, such models often lack transparency in terms of feature selection and weighting, making it difficult for external validation and traceability of these tools. Additionally, many algorithms were developed using small, homogeneous populations, limiting their external validity in real-world use.

A further limitation is the cross-sectional or retrospective design of most included studies, as longitudinal data are essential to determine whether wearable-derived BP trends are predictive of incident cardiovascular events, rather than simply correlating with known risk factors.

### Clinical usability

Despite technological advances, translation into clinical practice remains limited. One central issue is the need for calibration and, especially in ambulatory settings, frequent re-calibration [42]. This undermines their role as truly autonomous devices.

Moreover, motion sensitivity remains a major technical hurdle. Continuous BP measurement during daily activity introduces significant noise, and although studies incorporated accelerometer data or filtering algorithms to compensate [64], artefacts remain problematic—especially during physical activity or sleep [5, 58].

User-related factors also impact accuracy. Skin tone or tattoos affect PPG signal quality. Furthermore, comfort plays a crucial role in long-term adherence. Devices requiring firm skin contact, exact placement. or multiple sensors in complex locations are less likely to be used correctly and consistently, in the ambulatory setting. One approach to decrease the risk of sensor displacement is the implementation of a liquid-filled capsule, increasing contact area to the skin as exemplified by Fan et al.[14].

### Ethical, regulatory and societal considerations

As wearable devices become increasingly integrated into health monitoring, data privacy and ethical use gain importance. Most commercial devices rely on cloud-based storage and AI-driven analysis, creating potential vulnerabilities for data breaches, misuse by third parties (e.g., insurers) and loss of control by the user [23].

The interpretation of AI-derived risk scores for users with little medical knowledge and a lack of clinical context could lead to unnecessary anxiety or false reassurance. With the objective of achieving reliable risk assessments, aiming at relieving the burden on healthcare, manufacturers bear a great responsibility, as any misjudgement could have serious health consequences for the user.

From a regulatory standpoint, most devices reviewed are still considered lifestyle devices and are not certified for medical use. Formal clinical trials and approval processes are necessary to bridge this gap, but manufacturers often lack incentive or infrastructure to pursue such pathways.

### Implications for obstetrics and gynaecology practice

Cardiovascular risk prevention represents a life-course issue in women’s health, extending beyond traditional cardiologic care. Obstetrics and gynaecology provide unique and repeated points of contact with women during key cardiovascular risk transitions, including pregnancy, the postpartum period, and midlife.

Women with a history of hypertensive disorders during pregnancy, such as preeclampsia or gestational hypertension, carry an increased risk of developing chronic hypertension and cardiovascular disease later in life. However, structured long-term follow-up after pregnancy is often fragmented. Wearable blood pressure monitoring could, in the future, support extended postpartum surveillance by enabling low-threshold, longitudinal blood pressure tracking beyond the immediate postnatal period.

In outpatient gynaecology settings, particularly menopause and midlife clinics, wearable blood pressure monitoring may offer an opportunity to detect masked or evolving hypertension in women who otherwise do not undergo regular cardiovascular screening. Continuous or repeated measurements may help identify early risk patterns that are not captured by sporadic office-based assessments.

Importantly, the role of wearable blood pressure devices in obstetrics and gynaecology should be viewed as complementary rather than diagnostic. Their potential lies in facilitating awareness, longitudinal risk stratification and timely referral to primary care or cardiology, thereby strengthening the role of gynaecology as a gateway to preventive cardiovascular care across the female life course.

### Limitations

This systematic review has several limitations. The review was deliberately conceived with a broad scope to capture the full technological spectrum of non-invasive, wearable BP monitoring and its potential application in cardiovascular risk prediction across disciplines.

Although the project initially aimed to jointly assess wearable technologies for both BP and lipid monitoring, the large volume of literature and the methodological diversity of both fields necessitated a subsequent division into two complementary reviews. This step was taken to ensure sufficient depth of analysis and methodological rigor.

A key limitation arises from the pronounced heterogeneity of the included studies, which differed substantially in sensor technology, validation methodology, reference standards, study populations and outcome measures. This heterogeneity precluded quantitative meta-analysis and limited direct comparability between studies. However, this variability also reflects the dynamic and exploratory nature of this rapidly evolving research field.

Most studies were conducted under controlled laboratory conditions, frequently in small cohorts of young and predominantly healthy participants. As a result, the transferability of accuracy estimates to real-world, ambulatory settings and to populations with hypertension or established cardiovascular disease remains limited and requires targeted future investigations.

Importantly, none of the included studies reported sex-disaggregated accuracy metrics, despite enrolling both female and male participants. This limitation does not necessarily indicate an absence of sex-related differences but rather highlights a critical research gap. Given the known sex-specific differences in cardiovascular physiology and the underdiagnosis of cardiovascular disease in women, future studies should explicitly address the sex-specific performance of wearable BP technologies.

Finally, most available studies applied cross-sectional or short-term validation designs. Longitudinal investigations assessing whether wearable-derived BP trajectories predict incident cardiovascular events are still largely lacking. Until such evidence becomes available, the true prognostic value of these technologies in clinical risk stratification remains to be determined.

Although many studies reported accuracy metrics such as mean absolute error, correlation coefficients or Bland–Altman limits of agreement, the substantial heterogeneity in sensor principles, calibration approaches, validation settings and reported endpoints precluded meaningful quantitative pooling. Any attempt at meta-analysis would therefore risk producing statistically misleading and clinically uninterpretable summary estimates.

## Conclusion

This systematic review provides a comprehensive overview of current wearable-based blood pressure (BP) monitoring technologies and their potential role in cardiovascular disease (CVD) risk prediction. Wearable BP monitoring has advanced considerably in recent years and represents a promising, non-invasive approach for continuous cardiovascular health assessment and primary prevention. Given that CVD remains underdiagnosed in women, reliable tools for early risk detection are of particular clinical relevance.

Across the reviewed studies, photoplethysmography-based devices were most frequently investigated and demonstrated substantial potential, especially when combined with machine learning algorithms for cardiovascular risk estimation. However, important challenges remain, including inconsistent accuracy for systolic BP, susceptibility to motion artefacts, the need for repeated calibration, and limited external validation of prediction models. Furthermore, most studies are still restricted to controlled laboratory settings, with a lack of robust long-term data from real-world outpatient environments and despite known sex-specific differences, no sex-disaggregated accuracy metrics were provided by the included papers. To reduce the existing gap in the evidence of women’s cardiovascular health, research focussing on women in particular is greatly needed.

Hypertension often remains asymptomatic for long periods and affects women across the lifespan. Consequently, early detection and primary prevention cannot rely solely on symptom-driven consultations or age-based routine check-ups. In clinical practice, gynaecologists are often the physicians who see women most regularly during their reproductive years and around menopause. Through opportunistic screening for risk factors such as hypertension, offering closer monitoring and facilitating timely referral to primary care or cardiology, gynaecologists may therefore have a substantial impact on long-term cardiovascular outcomes.

To fully realise the clinical potential of wearable-based BP monitoring for cardiovascular risk assessment—especially in women—future research must prioritise longitudinal, regulatory-grade validation studies in representative outpatient populations. Study cohorts should cover a broad range of age, comorbidities, and BP levels and must explicitly address sex-specific performance. Only through rigorous clinical validation and standardisation can wearable BP technologies evolve from lifestyle tools to reliable instruments for CVD prevention in women.

## Supplementary Information

Below is the link to the electronic supplementary material.

Supplementary file1 (DOCX 35 KB)

Supplementary file2 (PDF 110 KB)

Supplementary file3 (XLSX 176 KB)

Supplementary file4 (DOCX 39 KB)

Supplementary file5 (XLSX 14 KB)

## Data Availability

No datasets were generated or analysed during the current study.
