# Supplementary material for: Non-invasive blood pressure monitoring using wearables for cardiovascular risk assessment: a systematic review
Source: Arch Gynecol Obstet. 2026 Jan 16;313(1):46. doi: 10.1007/s00404-025-08301-2 (PMC12811358; doi:10.1007/s00404-025-08301-2)
Supplement: Supplementary file 1 — Supplementary file1 (DOCX 35 KB) [file 404_2025_8301_MOESM1_ESM.docx]

**Identification**

**Identification of studies via databases and registers**






**Screening**

Records screened

(n = 14,863 )






Reports excluded:

Wrong Topic (n = 623)

Wrong Language (n =13)

Wrong Indication (n = 4)

Wrong Method (n = 210)

Wrong Intervention (n = 34)

Wrong Outcomes (n = 38)

Wrong Study Design (n = 65)

Duplicate (n = 8)

Animal Testing (n = 1)

Wrong Population (n = 7)

Lack of Quality (n = 6)



**Included**

Studies included in review

Total number: (n = 282)

Blood pressure (n = 245)

Lipids (n = 37)

*Consider, if feasible to do so, reporting the number of records identified from each database or register searched (rather than the total number across all databases/registers).

**If automation tools were used, indicate how many records were excluded by a human and how many were excluded by automation tools.

Source: Page MJ, et al. BMJ 2021;372:n71. doi: 10.1136/bmj.n71.

This work is licensed under CC BY 4.0. To view a copy of this license, visit <https://creativecommons.org/licenses/by/4.0/>
