# Supplementary material for: Non-invasive blood pressure monitoring using wearables for cardiovascular risk assessment: a systematic review
Source: Arch Gynecol Obstet. 2026 Jan 16;313(1):46. doi: 10.1007/s00404-025-08301-2 (PMC12811358; doi:10.1007/s00404-025-08301-2)
Supplement: Supplementary file 2 — Supplementary file2 (PDF 110 KB) [file 404_2025_8301_MOESM2_ESM.pdf]

Supplementary file 2 _Search strategies

Non-Invasive Blood Pressure Monitoring Using Wearables for Cardiovascular Risk Assessment & Lipid Monitoring Using Non-invasive Measurement technologies and Machine Learning

- MEDLINE ALL (Ovid)
- Embase (Ovid)
- Cochrane Library (Wiley)
- Science Citation Index Expanded (SCIE) & Emerging Sources Citation Index (ESCI), Web of Science Core Collection (Clarivate)
- Scopus (Elsevier)
- Clinicaltrials.gov (National Library of Medicine)

Below shows the search strategies that were designed and executed. The last run search was on May 1st, 2024.

MEDLINE All (Ovid)

1 cardiovascular diseases/ or heart diseases/ or heart failure/ or coronary artery disease/ or exp Cholesterol/ or blood pressure determination/ or Blood Pressure/

2 ("cardio vascular*" or cardiovascular* or cardiometabolic* or "cardio metabolic*" or "heart disease*" or "heart failure*" or "coronary artery disease*" or (blood adj2 lipid*) or cholesterol* or triglyceride* or HDL or LDL or "non HDL" or nonHDL or "non LDL" or nonLDL or lipoprotein? or "blood pressure*").ti,ab.

3 1 or 2

4 risk/ or risk assessment/ or risk factors/ or risk adjustment/ or forecasting/ or early diagnosis/ or Prognosis/ or (predict* or calculat* or scor* or risk* or detect* or track* or monitor* or forecast* or "early warning?" or "early diagnosis" or prognosis).ti,ab.

5 3 and 4

6 exp Heart Disease Risk Factors/

7 5 or 6

8 wearable electronic devices/ or fitness trackers/ or Smartphone/ or Mobile Applications/

9 (wearables or biosensor* or "bio sensor*" or "biomedical sensor*" or "bio medical sensor*" or "body sensor*" or smartwatch* or "smart watch*" or "apple watch*" or "smart wristband*" or "smart wrist band*" or "fitness track*" or "activity track*" or "body mounted sensor*" or "sensor based" or "smart gadget*" or "monitoring gadget*" or "wearable belt*" or "smart gear" or "wearable gear" or (wearable* adj3 (device* or technolog* or sensor* or electronic* or clothing or data or application* or app? or consumer or monitor* or track*)) or (smart* adj2 (device* or clothing or app? or application* or wearable* or electronic* or sensor* or monitor* or technolog*)) or ((sensor* or sensing or "bio sensing") adj1 (device* or technolog*))).ti,ab.

10 8 or 9

11 7 and 10

12 (exp animals/ or animal experimentation/ or models, animal/ or exp plants/ or exp fungi/) not humans/

13 11 not 12

14 limit 13 to yr="2010-current"

Embase (Ovid)

1 cardiovascular disease/ or heart disease/ or heart failure/ or coronary artery disease/ or blood pressure/ or exp lipid blood level/ or exp cholesterol level/

2 ("cardio vascular*" or cardiovascular* or cardiometabolic* or "cardio metabolic*" or "heart disease*" or "heart failure*" or "coronary artery disease*" or (blood adj2 lipid*) or cholesterol* or triglyceride* or HDL or LDL or "non HDL" or nonHDL or "non LDL" or nonLDL or lipoprotein? or "blood pressure*").ti,ab.

Supplementary file 2 _Search strategies

Non-Invasive Blood Pressure Monitoring Using Wearables for Cardiovascular Risk Assessment & Lipid Monitoring Using Non-invasive Measurement technologies and Machine Learning

3 1 or 2

4 risk factor/ or risk/ or risk algorithm/ or risk assessment/ or health risk assessment/ or "prediction and forecasting"/ or forecasting/ or early diagnosis/ or prognosis/ or (predict* or calculat* or scor* or risk* or detect* or track* or monitor* or forecast* or "early warning?" or "early diagnosis" or prognosis).ti,ab.

5 3 and 4

6 cardiovascular risk factor/ or cardiometabolic risk factor/ or cardiometabolic risk/ or heart disease risk factor/ or coronary risk/ or cardiovascular risk/

7 5 or 6

8 wearable sensor/ or activity tracker/ or smart watch/ or smartphone/ or exp mobile application/ 40901

9 (wearables or biosensor* or "bio sensor*" or "biomedical sensor*" or "bio medical sensor*" or "body sensor*" or smartwatch* or "smart watch*" or "apple watch*" or "smart wristband*" or "smart wrist band*" or "fitness track*" or "body mounted sensor*" or "sensor based" or "smart gadget*" or "monitoring gadget*" or "wearable belt*" or "smart gear" or "wearable gear" or (wearable* adj3 (device* or technolog* or sensor* or electronic* or clothing or data or application* or app? or consumer or monitor* or track*)) or (smart* adj2 (device* or clothing or app? or application* or wearable* or electronic* or sensor* or monitor* or technolog*)) or ((sensor* or sensing or "bio sensing") adj1 (device* or technolog*))).ti,ab.

10 8 or 9

11 7 and 10

12 (exp animal/ or exp invertebrate/ or nonhuman/ or animal experiment/ or animal tissue/ or animal model/ or exp plant/ or exp fungus/) not (exp human/ or human tissue/)

13 11 not 12

14 limit 13 to yr="2010-current"

Cochrane Library (Wiley)

Cochrane Reviews & CENTRAL

#1 ((cardio NEXT vascular*) OR cardiovascular* OR cardiometabolic* OR (cardio NEXT metabolic*) OR (heart NEXT disease*) OR (heart NEXT failure*) OR ("coronary artery" NEXT disease*) OR (blood NEAR/2 lipid*) OR cholesterol* OR triglyceride* OR HDL OR LDL OR "non HDL" OR nonHDL OR "non LDL" OR nonLDL OR lipoprotein? OR (blood NEXT pressure*)):ti,ab

#2 (predict* OR calculat* OR scor* OR risk* OR detect* OR track* OR monitor* OR forecast* OR "early warning" OR "early warnings" OR "early diagnosis" OR prognosis):ti,ab

#3 (wearables OR biosensor* OR (bio NEXT sensor*) OR (biomedical NEXT sensor*) OR ("bio medical" NEXT sensor*) OR (body NEXT sensor*) OR smartwatch* OR (smart NEXT watch*) OR (apple NEXT watch*) OR (smart NEXT wristband*) OR (smart NEXT wrist NEXT band*) OR (fitness NEXT track*) OR (activity NEXT track*) OR ("body mounted" NEXT sensor*) OR "sensor based" OR (smart NEXT gadget*) OR (monitoring NEXT gadget*) OR (wearable NEXT belt*) OR "smart gear" OR "wearable gear" OR (wearable* NEAR/3 (device* OR technolog* OR sensor* OR electronic* OR clothing OR data OR application* OR app? OR consumer OR monitor* OR track*)) OR (smart* NEAR/2 (device* OR clothing OR app? OR

Supplementary file 2 _Search strategies

Non-Invasive Blood Pressure Monitoring Using Wearables for Cardiovascular Risk Assessment & Lipid Monitoring Using Non-invasive Measurement technologies and Machine Learning

application* OR wearable* OR electronic* OR sensor* OR monitor* OR technolog*)) OR ((sensor* OR sensing OR "bio sensing") NEAR/1 (device* OR technolog*))):ti,ab

#4 #1 AND #2 AND #3

Science Citation Index Expanded & Emerging Sources Citation Index, Web of Science Core Collection, (Clarivate)

1: TS=("cardio vascular*" OR cardiovascular* OR cardiometabolic* OR "cardio metabolic*" OR "heart disease*" OR "heart failure*" OR "coronary artery disease*" OR (blood NEAR/2 lipid*) OR cholesterol* OR triglyceride* OR HDL OR LDL OR "non HDL" OR nonHDL OR "non LDL" OR nonLDL OR lipoprotein$ OR "blood pressure*"

2: TS=(predict* OR calculat* OR scor* OR risk* OR detect* OR track* OR monitor* OR forecast* OR "early warning$" OR "early diagnosis" OR prognosis)

3: TS=(wearables OR biosensor* OR "bio sensor*" OR "biomedical sensor*" OR "bio medical sensor*" OR "body sensor*" OR smartwatch* OR "smart watch*" OR "apple watch*" OR "smart wristband*" OR "smart wrist band*" OR "fitness track*" OR "activity track*" OR "body mounted sensor*" OR "sensor based" OR "smart gadget*" OR "monitoring gadget*" OR "wearable belt*" OR "smart gear" OR "wearable gear" OR (wearable* NEAR/3 (device* OR technolog* OR sensor* OR electronic* OR clothing OR data OR application* OR app$ OR consumer OR monitor* OR track*)) OR (smart* NEAR/2 (device* OR clothing OR app$ OR application* OR wearable* OR electronic* OR sensor* OR monitor* OR technolog*)) OR ((sensor* OR sensing OR "bio sensing") NEAR/1 (device* OR technolog*)))

4: #3 AND #2 AND #1

5: TI=("animal study" OR "animal tissue" OR "animal tissues" OR "animal cell" OR "animal cells" OR "animal model" OR "animal models" OR "animal experimentation" OR "animal experiment" OR "animal research" OR "laboratory animal" OR "laboratory animals" OR "in vivo" OR invertebrates OR mice OR mouse OR murine OR rat OR rats OR rodent OR rodents OR rabbit OR rabbits OR hamster OR hamsters OR pig OR pigs OR piglet OR piglets OR swine OR bird OR birds OR fish OR zebrafish OR frog OR frogs OR cat OR cats OR dog OR dogs OR canine OR canines OR feline OR felines OR cow OR cows OR cattle OR bovine OR "nonhuman primates" OR "non human primates" OR "non human animal" OR "nonhuman animal" OR "non human animals" OR "nonhuman animals")

6: (#4) NOT #5

7: (#4) NOT #5 and 2023 or 2022 or 2021 or 2020 or 2019 or 2016 or 2018 or 2017 or 2015 or 2014 or 2013 or 2012 or 2011 or 2010 (Publication Years)

Supplementary file 2 _Search strategies

Non-Invasive Blood Pressure Monitoring Using Wearables for Cardiovascular Risk Assessment & Lipid Monitoring Using Non-invasive Measurement technologies and Machine Learning

Scopus (Elsevier)

TITLE-ABS({cardio vascular disease} OR {cardio vascular diseases} OR {cardiovascular disease} OR {cardiovascular diseases} OR {cardiometabolic disease} OR {cardiometabolic diseases} OR {cardio metabolic disease} OR {cardio metabolic diseases} OR {heart disease} OR {heart diseases} OR {heart failure} OR {heart failures} OR {coronary artery disease} OR {coronary artery diseases} OR {blood lipid} OR {blood lipids} OR {lipids in blood} OR {lipids in the blood} OR {cholesterol} OR {triglyceride} OR {triglycerides} OR {HDL} OR {LDL} OR {non HDL} OR {nonHDL} OR {non LDL} OR {nonLDL} OR {lipoprotein} OR {lipoproteins} OR {blood pressure} OR {blood pressures}) AND TITLE-ABS({predict} OR {prediction} OR {predictions} OR {predicting} OR {calculate} OR {calculating} OR {calculation} OR {calculations} OR {score} OR {scores} OR {scoring} OR {risk} OR {risks} OR {risk score} OR {risk scores} OR {risk assessment} OR {risk assessments} OR {detect} OR {detects} OR {detecting} OR {detection} OR {detections} OR {track} OR {tracks} OR {tracking} OR {monitoring} OR {monitors} OR {monitor} OR {forecast} OR {forecasting} OR {early warning} OR {early warnings} OR {early diagnosis} OR {prognosis}) AND TITLE-ABS({wearables} OR {biosensor} OR {biosensors} OR {biomedical sensor} OR {biomedical sensors} OR {bio medical sensor} OR {bio medical sensors} OR {body sensor} OR {body sensors} OR {smartwatch} OR {smartwatches} OR {smart watch} OR {smart watches} OR {apple watch} OR {apple watches} OR {smart wristband} OR {smart wristbands} OR {smart wrist band} OR {smart wrist bands} OR {fitness tracker} OR {fitness trackers} OR {fitness tracking} OR {activity tracker} OR {activity trackers} OR {activity tracking} OR {body mounted sensor} OR {body mounted sensors} OR {sensor based} OR {smart gadget} OR {smart gadgets} OR {monitoring gadget} OR {monitoring gadgets} OR {wearable belt} OR {wearable belts} OR {smart gear} OR {wearable gear} OR {wearable device} OR {wearable devices} OR {wearable technology} OR {wearable technologies} OR {sensor} OR {sensors} OR {smart sensor} OR {smart sensors} OR {wearable data} OR {wearable clothing} OR {wearable application} OR {wearable applications} OR {wearable app} OR {wearable apps} OR {wearable monitor} OR {wearable monitors} OR {smart device} OR {smart devices} OR {smart technology} OR {smart technologies} OR {smart clothing} OR {smart phone app} OR {smart phone apps} OR {smartphone app} OR {smartphone apps} OR {smart app} OR {smart apps} OR {biosensing technology} OR {biosensing technologies} OR {sensing technology} OR {sensing technologies} OR {biosensing device} OR {biosensing devices} OR {sensing device} OR {sensing devices}) AND NOT TITLE({animal study} OR {animal tissue} OR {animal tissues} OR {animal cell} OR {animal cells} OR {animal model} OR {animal models} OR {animal experimentation} OR {animal experiment} OR {animal research} OR {laboratory animal} OR {laboratory animals} OR {in vivo} OR {invertebrates} OR {mice} OR {mouse} OR {murine} OR {rat} OR {rats} OR {rodent} OR {rodents} OR {rabbit} OR {rabbits} OR {hamster} OR {hamsters} OR {pig} OR {pigs} OR {piglet} OR {piglets} OR {swine} OR {bird} OR {birds} OR {fish} OR {zebrafish} OR {frog} OR {frogs} OR {cat} OR {cats} OR {dog} OR {dogs} OR {canine} OR {canines} OR {feline} OR {felines} OR {cow} OR {cows} OR {cattle} OR {bovine} OR {nonhuman primates} OR {non human primates} OR {non human animal} OR {nonhuman animal} OR {non human animals} OR {nonhuman animals}) AND NOT INDEX(medline) AND (LIMIT-TO (PUBYEAR,2023) OR LIMIT-TO (PUBYEAR,2022) OR LIMIT-TO (PUBYEAR,2021) OR LIMIT-TO (PUBYEAR,2020) OR LIMIT-TO (PUBYEAR,2019) OR LIMIT-TO (PUBYEAR,2018) OR LIMIT-TO (PUBYEAR,2017) OR LIMIT-TO (PUBYEAR,2016) OR LIMIT-TO (PUBYEAR,2015) OR LIMIT-TO (PUBYEAR,2014) OR LIMIT-TO (PUBYEAR,2013) OR LIMIT-TO (PUBYEAR,2012) OR LIMIT-TO (PUBYEAR,2010))

Clinical Trials.gov (National Library of Medicine)

Wearables OR "smart device" OR "smart devices" OR "body sensor" OR "body sensors" OR "smart phone app" OR "smart phone apps" OR "smart watch" OR "smart watches" OR "activity tracker" OR "activity trackers" OR "fitness tracker" OR "fitness trackers" | "cardiovascular disease" OR "cardiovascular diseases" OR "cardiometabolic disease" OR "cardiometabolic diseases" OR "heart disease" OR "heart diseases" OR "blood pressure" OR "blood pressures" OR "blood lipid" OR "blood lipids"
