# Supplementary material for: Non-invasive blood pressure monitoring using wearables for cardiovascular risk assessment: a systematic review
Source: Arch Gynecol Obstet. 2026 Jan 16;313(1):46. doi: 10.1007/s00404-025-08301-2 (PMC12811358; doi:10.1007/s00404-025-08301-2)
Supplement: Supplementary file 4 — Supplementary file4 (DOCX 39 KB) [file 404_2025_8301_MOESM4_ESM.docx]

**REFERENCE LIST:**

Abiri A, Chou EF, Qian C, Rinehart J, Khine M. Intra-beat biomarker for accurate continuous non-invasive blood pressure monitoring. Sci. 2022;12(1):16772.

Ahmed N, Banerjee R, Ghose A, Sinharay A, editors. Feasibility analysis for estimation of blood pressure and heart rate using a smart eye wear2015.

Akbulut FP, Akan A. A smart wearable system for short-term cardiovascular risk assessment with emotional dynamics. Measurement. 2018;128:237–46.

Al-Qatatsheh A, Morsi Y, Zavabeti A, Zolfagharian A, Salim N, A ZK, et al. Blood Pressure Sensors: Materials, Fabrication Methods, Performance Evaluations and Future Perspectives. Sensors (Basel). 2020;20(16):11.

Al-Shaher MA, Al-Khafaji NJ, editors. E-healthcare system to monitor vital signs2017.

Almarshad MA, Islam MS, Al-Ahmadi S, BaHammam AS. Diagnostic Features and Potential Applications of PPG Signal in Healthcare: A Systematic Review. Healthcare. 2022;10(3):28.

Anisimov AA, Ananyeva A, editors. Development of system for noninvasive cuffless estimation of blood pressure2014.

Ankishan H, Ari F, Celik H, Frederick BB, Hocke LM, Erdogan SB, et al. A new system for cuffless blood pressure measurement. Appl Acoust. 2023;212.

Antsiperov VE, Mansurov GK. Arterial blood pressure monitoring by active sensors based on heart rate estimation and pulse wave pattern prediction. 2016;26(3):533–47.

Badran D, Abreu P, Restivo MT, editors. Blood Pressure Measurement2019.

Baek J, Kim J, Kim N, Lee D, Park SM, editors. Validation of Cuffless Blood Pressure Monitoring Using Wearable Device2019.

Ballinger B, Hsieh J, Singh A, Sohoni N, Wang J, Tison GH, et al., editors. Deepheart: Semi-supervised sequence learning for cardiovascular risk prediction2018.

Bekiri R, Djeffal A, Hettiri M, editors. A remote medical monitoring system based on data mining2020.

Beyaz MI. An acoustic blood pressure sensing scheme using time of flight and shear wave elastography techniques. Sens Actuator A-Phys. 2021;330:7.

Bijender, Kumar S, Soni A, Kumar A. Evaluation of blood pressure using a flexible and wearable capacitive pressure sensor. RSC Adv. 2023;13(50):35397–407.

Chen JW, Huang HK, Fang YT, Lin YT, Li SZ, Chen BW, et al. A Data-Driven Model with Feedback Calibration Embedded Blood Pressure Estimator Using Reflective Photoplethysmography. Sensors (Basel). 2022;22(5):27.

Chen S, Qi J, Fan S, Qiao Z, Yeo JC, Lim CT. Flexible Wearable Sensors for Cardiovascular Health Monitoring. Ad. 2021;10(17):e2100116.

Chino S, Ishizawa H, Hosoya S, Koyama S, Fujimoto K, Kawamura T, editors. Research for wearable multiple vital sign sensor using fiber Bragg Grating-Verification of several pulsate points in human body surface2017.

Cohen Z, Haxha S. Optical-Based Sensor Prototype for Continuous Monitoring of the Blood Pressure. IEEE Sens J. 2017;17(13):4258–68.

Dai BY, Gao CC, Xie YN. Flexible wearable devices for intelligent health monitoring. View-China.8.2022

Das M, Choudhary T, Sharma LN, Bhuyan MK. Noninvasive Accelerometric Approach for Cuffless Continuous Blood Pressure Measurement. IEEE Trans Instrum Meas. 2021;70:9.

De Marchi B, Frigerio M, De Nadai S, Longinotti-Buitoni G, Aliverti A. Blood Pressure Continuous Measurement through a Wearable Device: Development and Validation of a Cuffless Method. Sensors. 2021;21(21):20.

Demircik F, Lier A, Pfutzner J, Kessler K, Strobl S, Pfutzner A. A non-invasive fingertip-mounted device to measure multiple physiological bio-parameters for disease management improvement. 2018;12(2):A17.

Dias D, Paulo Silva Cunha J. Wearable Health Devices-Vital Sign Monitoring, Systems and Technologies. Sensors (Basel). 2018;18(8):25.

Ding X, Dai W, Luo N, Liu J, Zhao N, Zhang Y, editors. A flexible tonoarteriography-based body sensor network for cuffless measurement of arterial blood pressure2015.

Dinh A, Luu L, Cao T, editors. Blood pressure measurement using finger ECG and photoplethysmogram for IoT2018.

Effil NJ, Rajeswari R. Wavelet scattering transform and long short-term memory network-based noninvasive blood pressure estimation from photoplethysmograph signals. Signal Image Video Process. 2022;16(1):1–9.

Fan XY, Huang Y, Ding XR, Luo NQ, Li CL, Zhao N, et al. Alignment-Free Liquid-Capsule Pressure Sensor for Cardiovascular Monitoring. Adv Funct Mater. 2018;28(44):9.

Fang L, Chen X, Fang Z, Tong K, Liu J, He Z, et al., editors. Multi-parameter health monitoring watch2017.

Farman A., El-Sappagh S, Islam SMR, Kwak D, Ali A, Imran M, et al. A smart healthcare monitoring system for heart disease prediction based on ensemble deep learning and feature fusion. Inf Fusion. 2020;63:208–22.

Florez D, Sepulveda J, editors. BlooXY: On a non-invasive blood monitor for the IoT context2017.

Fortin J, Rogge DE, Fellner C, Flotzinger D, Grond J, Lerche K, et al. A novel art of continuous noninvasive blood pressure measurement. Nat Commun. 2021;12(1):1387.

Gepner Y, Nachman D, Gilan A, Goldstein N, Constantini K, Littman R, et al. Using a novel non-invasive, cuff-less, wireless device for 24-hour ambulatory blood pressure monitoring. 2021;39(SUPPL 1):e135.

Gholamhosseini H, Biswas J, Zhang H, Jayachandran M, Baig MM, editors. Cuff-less, non-invasive and continuous blood pressure monitoring using indirect methods2015.

Ghosh S, Banerjee A, Ray N, Wood PW, Boulanger P, Padwal R, editors. Using Accelerometric and Gyroscopic Data to Improve Blood Pressure Prediction from Pulse Transit Time Using Recurrent Neural Network2018.

Ghosh S, Chattopadhyay BP, Roy RM, Mukherjee J, Mahadevappa M. Non-invasive cuffless blood pressure and heart rate monitoring using impedance cardiography. Intell Med. 2022;2(4):199–208.

Gircys R, Kazanavicius E, Maskeliunas R, Damasevicius R, Wozniak M. Wearable system for real-time monitoring of hemodynamic parameters: Implementation and evaluation. 2020;59 (no pagination).

Gircys R, Liutkevicius A, Kazanavicius E, Lesauskaite V, Damuleviciene G, Janaviciute A. Photoplethysmography-Based Continuous Systolic Blood Pressure Estimation Method for Low Processing Power Wearable Devices. Appl Sci-Basel. 2019;9(11):16.

Gogiberidze N, Suvorov A, Sultygova E, Sagirova Z, Kuznetsova N, Gognieva D, et al. Practical Application of a New Cuffless Blood Pressure Measurement Method. 2023;30(4):586–98.

Gomes E, Naima R, Liao C, Shay O. Waveform Morphology Comparison in Wearable Blood Pressure Sensors. Annu Int Conf IEEE Eng Med Biol Soc. 2022;2022:2902–5.

Gong S, Yap LW, Zhang Y, He J, Yin J, Marzbanrad F, et al. A gold nanowire-integrated soft wearable system for dynamic continuous non-invasive cardiac monitoring. Biosens Bioelectron. 2022;205:114072.

Guo CY, Chang CC, Wang KJ, Hsieh TL. Assessment of a Calibration-Free Method of Cuffless Blood Pressure Measurement: A Pilot Study. 2023;11:318–29.

Guo Y, Liu X, Peng S, Jiang X, Xu K, Chen C, et al. A review of wearable and unobtrusive sensing technologies for chronic disease management. Comput Biol Med. 2021;129:104163.

Gupta A, Yadav S, Shahid S, Venkanna U, editors. HeartCare: IoT based heart disease prediction system2019.

Gupta S, Singh A, Sharma A, editors. PPG-based End-to-End Framework for Cuffless Blood Pressure Estimation Using CNN-LSTM2023: Institute of Electrical and Electronics Engineers Inc.

Han M, Lee YR, Park T, Ihm SH, Pyun WB, Burkard T, et al. Feasibility and measurement stability of smartwatch-based cuffless blood pressure monitoring: A real-world prospective observational study. Hypertens Res. 2023;46(4):922–31.

Heimark S, Botker-Rasmussen KG, Rindal OMH, Seeberg TM, Stepanov A, Boysen ES, et al. Comparison of Continuous Mean Arterial Pressure by a Wearable Cuff-Less Device against Intra-Arterial Measurements. 2022;40(Supplement 1):e107–e8.

Heimark S, Hove C, Stepanov A, Boysen ES, Gloersen O, Botke-Rasmussen KG, et al. Accuracy and User Acceptability of 24-hour Ambulatory Blood Pressure Monitoring by a Prototype Cuffless Multi-Sensor Device Compared to a Conventional Oscillometric Device. Blood Press. 2023;32(1):2274595.

Heravi MAY, Khalilzadeh MA. Designing and constructing an optical system to measure continuous and cuffless blood pressure using two pulse signals. 2014;10-11(1-4):215–33.

Hoang Long NM, Kim JJ, Chung WY. A Prototype Wristwatch Device for Monitoring Vital Signs Using Multi-wavelength Photoplethysmography Sensors. 2021. p. 312–8.

Holyoke P, Yogaratnam K, Kalles E. Web-Based Smartphone Algorithm for Calculating Blood Pressure From Photoplethysmography Remotely in a General Adult Population: Validation Study. J Med Internet Res. 2021;23(4):e19187.

Honcharuk A, Adamenko Y, editors. Portable device for monitoring blood pressure2019.

Hosanee M, Chan G, Welykholowa K, Cooper R, Kyriacou PA, Zheng D, et al. Cuffless Single-Site Photoplethysmography for Blood Pressure Monitoring. J. 2020;9(3):07.

Hsiao CY, Han CF, Lee RG, Hsiao CC, Lin R, editors. Feasibility Study of Dual-PPG Sensors for Blood Velocity and Pressure Estimation2019.

Hsu YP, Young DJ, editors. Skin-surface-coupled personal health monitoring system2013.

Huang KH, Tan F, Wang TD, Yang YJ, editors. A Blood Pressure Monitoring Device with Tactile and Tension Sensors Assisted by a Machine Learning Technique2019.

Huang SC, Hung PH, Hong CH, Wang HM. A New Image Blood Pressure Sensor Based on PPG, RRT, BPTT, and Harmonic Balancing. IEEE Sens J. 2014;14(10):3685–92.

Huynh TH, Jafari R, Chung WY. Noninvasive Cuffless Blood Pressure Estimation Using Pulse Transit Time and Impedance Plethysmography. IEEE Trans Biomed Eng. 2019;66(4):967–76.

Ibrahim B, Jafari R. Continuous Blood Pressure Monitoring using Wrist-worn Bio-impedance Sensors with Wet Electrodes. IEEE Biomed Circuits Syst Conf. 2018.

Ibrahim B, Jafari R. Cuffless Blood Pressure Monitoring from an Array of Wrist Bio-Impedance Sensors Using Subject-Specific Regression Models: Proof of Concept. IEEE trans. 2019;13(6):1723–35.

Ibrahim B, Jafari R. Cuffless blood pressure monitoring from a wristband with calibration-free algorithms for sensing location based on bio-impedance sensor array and autoencoder. Sci. 2022;12(1):319.

Islam MA, Ahmad M, editors. Design and implementation of non-invasive continuous blood pressure measurement and monitoring system using photoplethysmography2019.

Islam MM, Rafi FHM, Mitul AF, Ahmad M, Rashid MA, Malek MFBA, editors. Development of a noninvasive continuous blood pressure measurement and monitoring system2012.

Islam SMS, Chow CK, Daryabeygikhotbehsara R, Subedi N, Rawstorn J, Tegegne T, et al. Wearable cuffless blood pressure monitoring devices: a systematic review and meta-analysis. Eur. 2022;3(2):323–37.

Islam SMS, Maddison R. A Comparison of Blood Pressure Data Obtained From Wearable, Ambulatory, and Home Blood Pressure Monitoring Devices: Prospective Validation Study. J Med Internet Res. 2020;22(11):7.

Ismail SNA, Nayan NA, Mohammad Haniff MAS, Jaafar R, May Z. Wearable Two-Dimensional Nanomaterial-Based Flexible Sensors for Blood Pressure Monitoring: A Review. Nanomaterials (Basel). 2023;13(5):24.

Jegan R, Jose PSH, Rajalakshmy P, Raj PAC, Kanmani HJ, Nimi WS. Methodological Role of Mathematics to Estimate Human Blood Pressure through Biosensors. Int J Integr Eng. 2021;13(1):240–8.

Jenifer M, Charlyn Pushpa Latha G, Rinesh S, Thamaraiselvi K, editors. Internet of Things (IOT) based Patient health care Monitoring System using electronic gadget2022.

Jeong IC, Bychkov D, Searson PC. Wearable Devices for Precision Medicine and Health State Monitoring. IEEE Trans Biomed Eng. 2019;66(5):1242–58.

Jing L, Yuan-Ting Z, Xiao-Rong D, Wen-Xuan D, Ni Z. A preliminary study on multi-wavelength PPG based pulse transit time detection for cuffless blood pressure measurement. Annu Int Conf IEEE Eng Med Biol Soc. 2016;2016:615–8.

Kanai D, Wakui H, Sato S, Lee J, Urate S, Tanaka S, et al. Comparing Blood Pressure Measurements between a Novel Cuff-Less Earphone-Type Device Based on Green Reflected Photoplethysmography and a Standard Cuff-Based Sphygmomanometer. 2023;41(Supplement 1):e204–e5.

Kao YH, Chao PCP, Wey CL, editors. A continuous opto-electronic sensor for blood pressure monitoring with real-time system2017.

Karatzia L, Aung N, Aksentijevic D. Artificial intelligence in cardiology: Hope for the future and power for the present. Front. 2022;9:945726.

Kario K. Management of Hypertension in the Digital Era: Small Wearable Monitoring Devices for Remote Blood Pressure Monitoring. Hypertension. 2020;76(3):640–50.

Katsuragawa Y, Ishizawa H, editors. Non-invasive blood pressure measurement by pulse wave analysis using FBG sensor2015.

Kaur H, Bhutto JK, Choudhary BS. Methods for continuous non-invasive measurement of blood pressure: Pulse transit time a review. 2018;12:S863–S72.

Kaur P, Saini HS, Kaur B. Wearable sensors for monitoring vital signs of patients. 2018;7(2):62–5.

Kaushik P, Sethi P, editors. A comprehensive study on blood pressure measurement techniques2018.

Khan Mamun MMR, Sherif A. Advancement in the Cuffless and Noninvasive Measurement of Blood Pressure: A Review of the Literature and Open Challenges. Bioengineering (Basel). 2022;10(1):24.

Khedraki R, Srivastava AV, Bhavnani SP. Framework for Digital Health Phenotypes in Heart Failure: From Wearable Devices to New Sensor Technologies. Heart Fail Clin. 2022;18(2):223–44.

Kim DK, Kim YT, Kim H, Kim DJ. DeepCNAP: A Deep Learning Approach for Continuous Noninvasive Arterial Blood Pressure Monitoring Using Photoplethysmography. IEEE j. 2022;26(8):3697–707.

Kim J, Chou EF, Le J, Wong S, Chu M, Khine M. Soft Wearable Pressure Sensors for Beat-to-Beat Blood Pressure Monitoring. Ad. 2019;8(13):e1900109.

Kim Y, Lee J, editors. Cuffless and non-invasive estimation of a continuous blood pressure based on PTT2010.

Koiv H, Rist M, Min M. Development of bioimpedance sensing device for wearable monitoring of the aortic blood pressure curve. tm-Tech Mess. 2018;85(5):366–77.

Konstantinidis D, Iliakis P, Tatakis F, Thomopoulos K, Dimitriadis K, Tousoulis D, et al. Wearable blood pressure measurement devices and new approaches in hypertension management: the digital era. J Hum Hypertens. 2022;23:23.

Krittanawong C, Rogers AJ, Johnson KW, Wang Z, Turakhia MP, Halperin JL, et al. Integration of novel monitoring devices with machine learning technology for scalable cardiovascular management. Nat Rev Cardiol. 2021;18(2):75–91.

Ku CJ, Wang Y, Chang CY, Wu MT, Dai ST, Liao LD. Noninvasive blood oxygen, heartbeat rate, and blood pressure parameter monitoring by photoplethysmography signals. Heliyon. 2022;8(11):e11698.

Kumar NV, Pant S, Sridhar S, Marulasiddappa V, Srivatzen S, Asokan S. Fiber Bragg Grating-Based Pulse Monitoring Device for Real-Time Non-Invasive Blood Pressure Measurement - A Feasibility Study. 2021;21(7):9179–85.

Kumar S, Victoria-Castro AM, Melchinger H, O'Connor KD, Psotka M, Desai NR, et al. Wearables in Cardiovascular Disease. J Cardiovasc Transl Res.12.2022

Kumar S, Yadav S, Kumar A. Blood pressure measurement techniques, standards, technologies, and the latest futuristic wearable cuff-less know-how. Sens Diagn. 2024;3(2):181–202.

Kumar VS, Krishnamoorthi C. Development of electrical transduction based wearable tactile sensors for human vital signs monitor: Fundamentals, methodologies and applications. Sens Actuator A-Phys. 2021;321:34.

Kusumah IH, Artiyasa M, Al B, Khoiri MI, Ramadhan AD, Supiyandi, editors. Blood Pressure Measurement using in Wrist PPG and ECG2020.

Kuwabara M, Harada K, Hishiki Y, Kario K. Validation of two watch-type wearable blood pressure monitors according to the ANSI/AAMI/ISO81060-2:2013 guidelines: Omron HEM-6410T-ZM and HEM-6410T-ZL. J Clin Hypertens (Greenwich). 2019;21(6):853–8.

Kuzmanov I, Bogdanova AM, Kostoska M, Ackovska N, editors. Fast Cuffless Blood Pressure Classification with ECG and PPG signals using CNN-LSTM Models in Emergency Medicine2022.

Lahil NIFM, Wong F, Chekima A, Abang S, Besar NA, Kiring AJ, et al., editors. Non-Invasive Blood Pressure and Heart Rate Sensing Using Photoplethysmogram Sensor2021.

Lazazzera R, Belhaj Y, Carrault G. A New Wearable Device for Blood Pressure Estimation Using Photoplethysmogram. Sensors (Basel). 2019;19(11):04.

Lazazzera R, Carrault G. MonEco: a Novel Health Monitoring Ecosystem to Predict Respiratory and Cardiovascular Disorders. 2023;44(2) (no pagination).

Lee K, Chae HY, Park K, Lee Y, Cho S, Ko H, et al. A Multi-Functional Physiological Hybrid-Sensing E-Skin Integrated Interface for Wearable IoT Applications. IEEE trans. 2019;13(6):1535–44.

Lee KM, Qian Z, Yabuki R, Du B, Kino H, Fukushima T, et al., editors. Continuous Peripheral Blood Pressure Measurement with ECG and PPG Signals at Fingertips2018.

Li H, Li A, Zhou Z, Jiang B, Yang Q, Liu X, et al., editors. Wearable Pulse Wave Sensor and Interface for Real-Time Dynamic Blood Pressure Monitoring2021.

Li L, Liu Y, Sheng S, Song C, Fan W, Sun Q, editors. Wearable Smartwatch based on Optical Fiber for Continuous Blood Pressure Monitoring2016.

Li LY, Li YP, Yang LY, Fang F, Yan ZJ, Sun QZ. Continuous and Accurate Blood Pressure Monitoring Based on Wearable Optical Fiber Wristband. IEEE Sens J. 2021;21(3):3049–57.

Li LY, Liu YF, Song CY, Sheng SF, Yang LY, Yan ZJ, et al. Wearable Alignment-Free Microfiber-Based Sensor Chip for Precise Vital Signs Monitoring and Cardiovascular Assessment. Adv Fiber Mater. 2022;4(3):475–86.

Li LY, Sheng SF, Liu YF, Wen JP, Song CY, Chen ZP, et al. Automatic and continuous blood pressure monitoring via an optical-fiber-sensor-assisted smartwatch. PhotoniX. 2023;4(1):14.

Li S, Park Y, Luan H, Wang H, Kwon K, Rogers JA, et al. Measurement of blood pressure via a skin-mounted, non-invasive pressure sensor. 2021;88(10).

Li S, Wang H, Ma W, Qiu L, Xia K, Zhang Y, et al. Monitoring blood pressure and cardiac function without positioning via a deep learning-assisted strain sensor array. Sci Adv. 2023;9(32):eadh0615.

Li WD, Ke K, Jia J, Pu JH, Zhao X, Bao RY, et al. Recent Advances in Multiresponsive Flexible Sensors towards E-skin: A Delicate Design for Versatile Sensing. Small. 2022;18(7):e2103734.

Li, X. (1)

1. Zhou ZB, Cui TR, Li D, Jian JM, Li Z, Ji SR, et al. Wearable Continuous Blood Pressure Monitoring Devices Based on Pulse Wave Transit Time and Pulse Arrival Time: A Review. Materials (Basel). 2023;16(6):07.

Li, X. S. (1)

1. Meng KY, Chen J, Li XS, Wu YF, Fan WJ, Zhou ZH, et al. Flexible Weaving Constructed Self-Powered Pressure Sensor Enabling Continuous Diagnosis of Cardiovascular Disease and Measurement of Cuffless Blood Pressure. Adv Funct Mater. 2019;29(5):10.

Li, Y. (3)

1. Liu ZD, Li Y, Zhang YT, Zeng J, Chen ZX, Cui ZW, et al. Cuffless Blood Pressure Measurement Using Smartwatches: A Large-Scale Validation Study. 2023;27(9):4216–27.

2. Qin C, Li Y, Liu C, Ma X. Cuff-Less Blood Pressure Prediction Based on Photoplethysmography and Modified ResNet. Bioengineering (Basel). 2023;10(4):24.

3. Zhang J, Wu D, Li Y. Cuff-less and Calibration-free Blood Pressure Estimation Using Convolutional Autoencoder with Unsupervised Feature Extraction. Annu Int Conf IEEE Eng Med Biol Soc. 2019;2019:3323–6.

Li, Y. F. (1)

1. Lin J, Fu RM, Zhong XX, Yu P, Tan GX, Li W, et al. Wearable sensors and devices for real-time cardiovascular disease monitoring. Cell Rep Phys Sci. 2021;2(8):25.

Li, Y. P. (1)

1. Li LY, Li YP, Yang LY, Fang F, Yan ZJ, Sun QZ. Continuous and Accurate Blood Pressure Monitoring Based on Wearable Optical Fiber Wristband. IEEE Sens J. 2021;21(3):3049–57.

Liao C, Shay O, Gomes E, Bikhchandani N, editors. Noninvasive continuous blood pressure measurement with wearable millimeter wave device2021.

Lin BH, Ma ZC, Atef M, Ying L, Wang GX. Low-Power High-Sensitivity Photoplethysmography Sensor for Wearable Health Monitoring System. IEEE Sens J. 2021;21(14):16141–51.

Lin J, Fu RM, Zhong XX, Yu P, Tan GX, Li W, et al. Wearable sensors and devices for real-time cardiovascular disease monitoring. Cell Rep Phys Sci. 2021;2(8):25.

Liu J, Qiu S, Luo N, Lau SK, Yu H, Kwok T, et al. PCA-Based Multi-Wavelength Photoplethysmography Algorithm for Cuffless Blood Pressure Measurement on Elderly Subjects. IEEE j. 2021;25(3):663–73.

Liu J, Yan BP, Zhang YT, Ding XR, Su P, Zhao N. Multi-Wavelength Photoplethysmography Enabling Continuous Blood Pressure Measurement With Compact Wearable Electronics. IEEE Trans Biomed Eng. 2019;66(6):1514–25.

Liu J, Zhang YT. Interoperability of wearable cuffless BP measuring devices. Annu Int Conf IEEE Eng Med Biol Soc. 2014;2014:1374–7.

Liu SH, Cai GH, Cheng DC, Huang YF, editors. Using impedance-plethysmography technique for cuffless blood pressure measurement2017.

Liu SH, Lai SH, Wang JJ, Tan TH, Huang YF. The Cuffless Blood Pressure Measurement with Multi-dimension Regression Model based on Characteristics of Pulse Waveform. Annu Int Conf IEEE Eng Med Biol Soc. 2019;2019:6838–41.

Lo Presti D, Massaroni C, Leitao CSJ, Domingues MD, Sypabekova M, Barrera D, et al. Fiber Bragg Gratings for Medical Applications and Future Challenges: A Review. IEEE Access. 2020;8:156863–88.

Liu J, Qiu S, Luo N, Lau SK, Yu H, Kwok T, et al. PCA-Based Multi-Wavelength Photoplethysmography Algorithm for Cuffless Blood Pressure Measurement on Elderly Subjects. IEEE j. 2021;25(3):663–73.

Luo NQ, Dai WX, Li CL, Zhou ZQ, Lu LY, Poon CCY, et al. Flexible Piezoresistive Sensor Patch Enabling Ultralow Power Cuffless Blood Pressure Measurement. Adv Funct Mater. 2016;26(8):1178–87.

Lyu QX, Gong S, Yin JL, Dyson JM, Cheng WL. Soft Wearable Healthcare Materials and Devices. Adv Healthc Mater. 2021;10(17):34.

Maduri PK, Dewangan Y, Yadav D, Chauhan S, Singh K, editors. IOT Based Patient Health Monitoring Portable Kit2020.

Mahrova V, Prochazka M, Penhaker M, editors. A Cardiovascular Model for the Development of Mathematical Algorithms for Non-invasive Continuous Blood Pressure Determination2022: Institute of Electrical and Electronics Engineers Inc.

Medina A, Lopez N, Galdos J, Supo E, Rendulich J, Sulla E. Continuous Blood Pressure Estimation in Wearable Devices Using Photoplethysmography: A Review. Int J Emerg Technol Adv Eng. 2022;12(10):104–13.

Mejía-Mejía E, Budidha K, Kyriacou PA, Mamouei M. Comparison of pulse rate variability and morphological features of photoplethysmograms in estimation of blood pressure. 2022;78.

Melville S, Teskey R, Philip S, Simpson JA, Lutchmedial S, Brunt KR. A Comparison and Calibration of a Wrist-Worn Blood Pressure Monitor for Patient Management: Assessing the Reliability of Innovative Blood Pressure Devices. J Med Internet Res. 2018;20(4):12.

Mena LJ, Felix VG, Ostos R, Gonzalez AJ, Martinez-Pelaez R, Melgarejo JD, et al. Mobile Personal Health Care System for Noninvasive, Pervasive, and Continuous Blood Pressure Monitoring: Development and Usability Study. JMIR Mhealth Uhealth. 2020;8(7):e18012.

Meng KY, Chen J, Li XS, Wu YF, Fan WJ, Zhou ZH, et al. Flexible Weaving Constructed Self-Powered Pressure Sensor Enabling Continuous Diagnosis of Cardiovascular Disease and Measurement of Cuffless Blood Pressure. Adv Funct Mater. 2019;29(5):10.

Meng XY, Zhang JL, Liu Y, Wen JB, Huang SL, editors. Design of electronic blood pressure monitoring system based on double filter2015.

Mia MMH, Mahfuz N, Habib MR, Hossain R, editors. An Internet of Things Application on Continuous Remote Patient Monitoring and Diagnosis2021.

Mieloszyk R, Twede H, Lester J, Wander J, Basu S, Cohn G, et al. A Comparison of Wearable Tonometry, Photoplethysmography, and Electrocardiography for Cuffless Measurement of Blood Pressure in an Ambulatory Setting. IEEE j. 2022;26(7):2864–75.

Miyauchi Y, Ishizawa H, Koyama S, Sato S, editors. Verification of the systolic blood-pressure measurement principle by FBG sensors2012.

Mohamed MB, Meddeb-Makhlouf A, Fakhfakh A, Kanoun O. Secure and Reliable ML-based Disease Detection for a Medical Wireless Body Sensor Networks. 2022;16:196–206.

Mohammadzadeh N, Gholamzadeh M, Saeedi S, Rezayi S. The application of wearable smart sensors for monitoring the vital signs of patients in epidemics: a systematic literature review. J Ambient Intell Humaniz Comput. 2020:1–15.

Mohd Sani MI, Abdullah NAS, Mohd Rosli M. Review on hypertension diagnosis using expert system and wearable devices. 2022;12(3):3166–75.

Momynaliev KT, Ivanov IV. Portable health monitoring devices. 2023;57(4):295–9.

Moon JH, Kang MK, Choi CE, Min J, Lee HY, Lim S. Validation of a wearable cuff-less wristwatch-type blood pressure monitoring device. Sci. 2020;10(1):19015.

Mujawar MS, Salunke D, Mulani D, Gajare A, Deshmukh PM, Ranjan NM, et al., editors. Smart Watch Assisted Multi-disease Detection Using Machine Learning: A Comprehensive Survey2024: Springer Science and Business Media Deutschland GmbH.

Munnoch R, Jiang P, editors. A personal medical device for multi-sensor, remote vital signs collection in the elderly2015.

Myint C, Lim KH, Wong KI, Gopalai AA, Oo MZ, editors. Blood Pressure measurement from Photo-Plethysmography to Pulse Transit Time2014.

Nachman D, Gilan A, Goldstein N, Constantini K, Littman R, Eisenkraft A, et al. Twenty-Four-Hour Ambulatory Blood Pressure Measurement Using a Novel Noninvasive, Cuffless, Wireless Device. 2021;34(11):1171–80.

Nakamura M, Nakamura J, Lopez G, Shuzo M, Yamada I. Collaborative processing of wearable and ambient sensor system for blood pressure monitoring. Sensors (Basel). 2011;11(7):6760–70.

Narasimhan R, Parlikar T, Verghesel G, McConnell MV. Finger-Wearable Blood Pressure Monitor. Annu Int Conf IEEE Eng Med Biol Soc. 2018;2018:3792–5.

Nidigattu GR, Mattela G, Jana S, editors. Non-invasive modeling of heart rate and blood pressure from a photoplethysmography by using machine learning techniques2020.

Noh S, Yoon C, Hyun E, Yoon HN, Chung TJ, Park KS, et al. Ferroelectret film-based patch-type sensor for continuous blood pressure monitoring. 2014;50(3):143–4.

Osman D, Jankovic M, Sel K, Pettigrew RI, Jafari R. Blood Pressure Estimation using a Single Channel Bio-Impedance Ring Sensor. Annu Int Conf IEEE Eng Med Biol Soc. 2022;2022:4286–90.

Paliakaite B, Charlton PH, Rapalis A, Plusciauskaite V, Piartli P, Kaniusas E, et al., editors. Blood Pressure Estimation Based on Photoplethysmography: Finger Versus Wrist2021.

Pan LYM, Han LY, Liu HX, Zhao JJ, Dong Y, Wang XH. Flexible sensor based on Hair-like microstructured ionic hydrogel with high sensitivity for pulse wave detection. Chem Eng J. 2022;450:10.

Pandit JA, Lores E, Batlle D. Cuffless Blood Pressure Monitoring: Promises and Challenges. Clin J Am Soc Nephrol. 2020;15(10):1531–8.

Pang YN, Liu B, Liu J, Wan SP, Wu T, Yuan J, et al. Singlemode-Multimode-Singlemode Optical Fiber Sensor for Accurate Blood Pressure Monitoring. 2022;40(13):4443–50.

Panula T, Sirkia JP, Kaisti M, editors. Control Method for Continuous Non-Invasive Arterial Pressure Monitoring using the Non-Pulsatile Component of the PPG Signal2021.

Panula T, Sirkia JP, Wong D, Kaisti M. Advances in non-invasive blood pressure measurement techniques. IEEE rev. 2022;11:11.

Park D, Cho SJ, Kim K, Woo H, Kim JE, Lee JY, et al. Prediction Algorithms for Blood Pressure Based on Pulse Wave Velocity Using Health Checkup Data in Healthy Korean Men: Algorithm Development and Validation. JMIR Med Inform. 2021;9(12):e29212.

Park J, Yang S, Sohn J, Lee J, Lee S, Ku Y, et al. Cuffless and Continuous Blood Pressure Monitoring Using a Single Chest-Worn Device. IEEE Access. 2019;7:135231–46.

Park SH, Choi SJ, Park KS. Advance continuous monitoring of blood pressure and respiration rate using denoising auto encoder and LSTM. Microsyst Technol.10.

Peng Y, Zhou J, Song X, Pang K, Samy A, Hao Z, et al. A Flexible Pressure Sensor with Ink Printed Porous Graphene for Continuous Cardiovascular Status Monitoring. Sensors (Basel). 2021;21(2):12.

Phan DT, Nguyen CH, Nguyen TDP, Tran LH, Park S, Choi J, et al. A Flexible, Wearable, and Wireless Biosensor Patch with Internet of Medical Things Applications. Biosensors (Basel). 2022;12(3):22.

Phan DT, Phan TTV, Huynh TC, Park S, Choi J, Oh J. Noninvasive, Wearable Multi Biosensors for Continuous, Long-term Monitoring of Blood Pressure via Internet of Things Applications. Comput Electr Eng. 2022;102:15.

Phillips SA, Ali M, Modrich C, Oke S, Elokda A, Laddu D, et al. Advances in Health Technology Use and Implementation in the Era of Healthy Living: Implications for Precision Medicine. 2019;62(1):44–9.

Pielmus AG, Klum M, Tigges T, Orglmeister R, Urban M. Progressive Dynamic Time Warping for Noninvasive Blood Pressure Estimation. 2020;6(3).

Pielmus AG, Klum M, Urban M. Single-signal noninvasive blood pressure estimation. 2020;65(SUPPL 1):S97.

Poli A, Cosoli G, Iadarola G, Spinsante S, Scalise L, editors. Feasibility of Blood Pressure Measurement through Wearable Devices: Analysis of Smartwatches Performance2022: Institute of Electrical and Electronics Engineers Inc.

Pour Ebrahim M, Heydari F, Wu T, Walker K, Joe K, Redoute JM, et al. Blood Pressure Estimation Using On-body Continuous Wave Radar and Photoplethysmogram in Various Posture and Exercise Conditions. Sci. 2019;9(1):16346.

Proença M, Falhi A, Ferrario D, Grossenbacher O, Porchet JA, Krauss J, et al. Continuous non-occlusive blood pressure monitoring at the sternum. 2012;57(SUPPL. 1 TRACK-F):2–5.

Qin C, Li Y, Liu C, Ma X. Cuff-Less Blood Pressure Prediction Based on Photoplethysmography and Modified ResNet. Bioengineering (Basel). 2023;10(4):24.

Qiu C, Wu T, Heydari F, Redoute JM, Yuce MR, editors. Wearable Blood Pressure Monitoring Based on Bio-Impedance and Photoplethysmography Sensors on the Arm2018.

Rachim VP, Chung WY. Multimodal Wrist Biosensor for Wearable Cuff-less Blood Pressure Monitoring System. Sci. 2019;9(1):7947.

Rachim VP, Huynh TH, Chung WY, editors. Wrist Photo-Plethysmography and Bio-Impedance Sensor for Cuff-Less Blood Pressure Monitoring2018.

Rachim VP, Kang S, Baek JH, Park SM. Unobtrusive, Cuffless Blood Pressure Monitoring Using a Soft Polymer Sensor Array With Flexible Hybrid Electronics. IEEE Sens J. 2021;21(8):10132–42.

Raji A, Golda Jeyasheeli P, Jenitha T, editors. IoT based classification of vital signs data for chronic disease monitoring2016.

Ran X, Luo FY, Lin ZM, Zhu ZY, Liu CJ, Chen B. Blood pressure monitoring via double sandwich-structured triboelectric sensors and deep learning models. Nano Res. 2022;15(6):5500–9.

Rasyid MUHA, Martono HY, Ariyadi BND, Nasution IN, editors. Design and integration of portable health sensors2021.

Rehman H, Kamal AK, Morris PB, Sayani S, Merchant AT, Virani SS. Mobile Health (mHealth) Technology for the Management of Hypertension and Hyperlipidemia: Slow Start but Loads of Potential. 2017;19(3) (no pagination).

Riaz F, Azad MA, Arshad J, Imran M, Hassan A, Rehman S. Pervasive blood pressure monitoring using Photoplethysmogram (PPG) sensor. Futur Gener Comp Syst. 2019;98:120–30.

Řiha K, Chmelař M, Čiž R. New ways of non-invasive measuring of blood circulation parameters. 2010;6(1):1–11.

Roos LG, Slavich GM. Wearable technologies for health research: Opportunities, limitations, and practical and conceptual considerations. 2023;113:444–52.

Samartkit P, Pullteap S. Non-invasive continuous blood pressure sensors in biomedical engineering research: A review. Sens Actuators A Phys. 2024;367.

Samartkit P, Pullteap S, Bernal O. A non-invasive heart rate and blood pressure monitoring system using piezoelectric and photoplethysmographic sensors. 2022;196.

Santo K, Redfern J. Digital Health Innovations to Improve Cardiovascular Disease Care. Curr Atheroscler Rep. 2020;22(12):71.

Savostin A, Tuleshov A, Koshekov K, Savostina G, Largin A. DEVISING A METHOD FOR PREDICTING A BLOOD PRESSURE LEVEL BASED ON ELECTROCARDIOGRAM AND PHOTOPLETHYSMOGRAM SIGNALS. East Eur J Enterp Technol. 2022;5(2-119):62–74.

Scardulla F, Montinaro N, D'Acquisto L, editors. Blood pressure acquisitions with a prototypal PPG-based device2022.

Schukraft S, Boukhayma A, Cook S, Caizzone A. Remote Blood Pressure Monitoring With a Wearable Photoplethysmographic Device (Senbiosys): Protocol for a Single-Center Prospective Clinical Trial. JMIR Res Protoc. 2021;10(10):e30051.

Schukraft S, Haddad S, Faucherre Y, Arroyo D, Togni M, Barison A, et al. Remote blood pressure monitoring with a wearable photoplethysmographic device in patients undergoing coronary angiography: the senbiosys substudy. Blood Press Monit. 2022;17:17.

Schutte AE, Kollias A, Stergiou GS. Blood pressure and its variability: classic and novel measurement techniques. Nat Rev Cardiol. 2022;19(10):643–54.

Sel K, Osman D, Huerta N, Edgar A, Pettigrew RI, Jafari R. Continuous cuffless blood pressure monitoring with a wearable ring bioimpedance device. npj digit. 2023;6(1):59.

Sheng H, Schwarz M, Borcsok J, editors. New concept to develop a safety sensor network for continuous noninvasive blood pressure monitoring2012.

Shi P, Xu Y, Yu H, editors. Design of Wearable System for Detection and Analysis of Physiological Information2017.

Silverio AA, Suarez CG, Silverio LAA, Dino JY, Duran JB, Catambing GEG. An Unobtrusive, Wireless and Wearable Single-Site Blood Pressure Monitor Based on an Armband Using Electrocardiography (ECG) and Reflectance Photoplethysmography (PPG) Signal Processing. Electronics. 2023;12(7):12.

Simjanoska M, Kochev S, Tanevski J, Bogdanova AM, Papa G, Eftimov T. Multi-level information fusion for learning a blood pressure predictive model using sensor data. Inf Fusion. 2020;58:24–39.

Singh B, Urooj S, Mishra S, Haldar S, editors. Blood pressure monitoring system using wireless technologies2019.

Singh H, Kumar D. Non-invasive cuffless blood pressure monitoring system. 2016. p. 174–94.

Slapnicar G, Lustrek M, Marinko M. Continuous Blood Pressure Estimation from PPG Signal. Inform-J Comput Inform. 2018;42(1):33–42.

Sola J, Proenca M, Chetelat O. Wearable PWV technologies to measure Blood Pressure: eliminating brachial cuffs. Annu Int Conf IEEE Eng Med Biol Soc. 2013;2013:4098–101.

Sola J, Vybornova A, Fallet S, Polychronopoulou E, Wurzner-Ghajarzadeh A, Wuerzne G. Validation of the Optical Aktiia Bracelet in Different Body Positions for the Persistent Monitoring of Blood Pressure. 2022;40(Supplement 1):e92.

Soon S, Svavarsdottir H, Downey C, Jayne DG. Wearable devices for remote vital signs monitoring in the outpatient setting: An overview of the field. 2020;6(2):55.

Stergiou GS. Are Cuffless Blood Pressure Measuring Devices Ready for Clinical Use? 2023;41(Supplement 1):e129.

Stergiou GS, Mukkamala R, Avolio A, Kyriakoulis KG, Mieke S, Murray A, et al. Cuffless blood pressure measuring devices: review and statement by the European Society of Hypertension Working Group on Blood Pressure Monitoring and Cardiovascular Variability. J Hypertens. 2022;40(8):1449–60.

Sun F, Zhao Z, Fang Z, Chen D, Chen X, Xuan Y, editors. Design and implementation of an ultra low power health monitoring node for wireless body sensor network2013.

TaheriNejad N, Rahmati Y. Blood Pressure Estimation Using a Single PPG Signal. 2021. p. 3–11.

Tan I, Gnanenthiran SR, Chan J, Kyriakoulis KG, Schlaich MP, Rodgers A, et al. Evaluation of the ability of a commercially available cuffless wearable device to track blood pressure changes. J Hypertens. 2023;41(6):1003–10.

Tan I, Gnanenthiran SR, Chan J, Kyriakoulis KG, Schlaich MP, Stergiou GS, et al. Cuffless Wearable 24-Hour Blood Pressure Monit Oring Versus Standard Ambulatory Monitoring. 2023;41(Supplement 1):e128.

Tao J, Qiu Y, Zhang J, Sun J, Liu Y, Wei C, et al. Improvement and Validation of a Novel Sphygmomanometer Based on Photoplethysmography. 2020;75(11):3578.

Tian S, Wang L, Zhu R. A flexible multimodal pulse sensor for wearable continuous blood pressure monitoring. Mater. 2024;05:05.

Tu TY, Chao PCP, Lee YP, Kao YH, editors. Optimal design and experimental validation of a new no-cuff blood pressure sensor based on a new finite element model2014.

Tu TY, Kao YH, Chao PCP, Lee YP, editors. Optimizing a new blood pressure sensor for maximum performance based on finite element model2014.

van Helmond N, Freeman CG, Hahnen C, Haldar N, Hamati JN, Bard DM, et al. The accuracy of blood pressure measurement by a smartwatch and a portable health device. Hosp Pract (Minneap). 2019;47(4):211–5.

Vaseekaran M, Kaese S, Gorlich D, Wiemer M, Samol A. WATCH-BPM-Comparison of a WATCH-Type Blood Pressure Monitor with a Conventional Ambulatory Blood Pressure Monitor and Auscultatory Sphygmomanometry. Sensors (Basel). 2023;23(21):31.

Vysotskaya N, Will C, Servadei L, Maul N, Mandl C, Nau M, et al. Continuous Non-Invasive Blood Pressure Measurement Using 60 GHz-Radar-A Feasibility Study. Sensors (Basel). 2023;23(8):19.

Wang L, Tian S, Zhu R. A new method of continuous blood pressure monitoring using multichannel sensing signals on the wrist. Microsyst. 2023;9:117.

Wang L, Xian H, Guo J, Li W, Wang J, Chen Q, et al. A novel blood pressure monitoring technique by smart HUAWEI WATCH: A validation study according to the ANSI/AAMI/ISO 81060-2:2018 guidelines. Front. 2022;9:923655.

Wang TW, Lin SF. Wearable Piezoelectric-Based System for Continuous Beat-to-Beat Blood Pressure Measurement. Sensors (Basel). 2020;20(3):05.

Wang TW, Syu JY, Chu HW, Sung YL, Chou L, Escott E, et al. Intelligent Bio-Impedance System for Personalized Continuous Blood Pressure Measurement. 2022;12(3) (no pagination).

Wang Y, Liu Z, Ma S. Cuff-less blood pressure measurement from dual-channel photoplethysmographic signals via peripheral pulse transit time with singular spectrum analysis. Physiol Meas. 2018;39(2):025010.

Wang YJ, Chen TY, Tsai MC, Wu CH. Noninvasive blood pressure monitor using strain gauges, a fastening band, and a wrist elasticity model. 2016;252:198–208.

Wang ZL, Zhao C, Qiu S. A system off human vita signs monitoring and activity recognition based on body sensor network. Sens Rev. 2014;34(1):42–50.

Watanabe N, Bando YK, Kawachi T, Yamakita H, Futatsuyama K, Honda Y, et al. Development and Validation of a Novel Cuff-Less Blood Pressure Monitoring Device. JACC Basic Transl Sci. 2017;2(6):631–42.

Welykholowa K, Hosanee M, Chan G, Cooper R, Kyriacou PA, Zheng D, et al. Multimodal Photoplethysmography-Based Approaches for Improved Detection of Hypertension. J. 2020;9(4):22.

Wen L, Dong S, Zhang Z, Gu C, Mao J, editors. Noninvasive Continuous Blood Pressure Monitoring Based on Wearable Radar Sensor with Preliminary Clinical Validation2022: Institute of Electrical and Electronics Engineers Inc.

Wu W, Zou Y, Tu C, Gao G, Chen Z, editors. Finger Versus Wrist Photoplethysmography Signals: Implications for Wearable Blood Pressure Monitoring2023: Institute of Electrical and Electronics Engineers Inc.

Xin Q, Wu J. A novel wearable device for continuous, non-invasion blood pressure measurement. Comput Biol Chem. 2017;69:134–7.

Yamakoshi T, Rolfe P, Yamakoshi KI. Cuffless blood pressure estimation based on haemodynamic principles: progress towards mobile healthcare. Peerj. 2021;9:e11479.

Yan J, Cai X, Zhu G, Guo R, Yan H, Wang Y. A non-invasive blood pressure prediction method based on pulse wave feature fusion. 2022;74 (no pagination).

Yang C, Shuo C, Tong W, Ting M. Novel blood pressure estimation method using single photoplethysmography feature. Annu Int Conf IEEE Eng Med Biol Soc. 2017;2017:1712–5.

Yang F, Xu W, Lyu W, Tan F, Yu C, Dong B. High Fidelity MZI-BCG Sensor With Homodyne Demodulation for Unobtrusive HR and BP Monitoring. 2022;22(8):7798–807.

Yang G, Pang G, Pang Z, Gu Y, Mantysalo M, Yang H. Non-Invasive Flexible and Stretchable Wearable Sensors With Nano-Based Enhancement for Chronic Disease Care. IEEE rev. 2019;12:34–71.

Yao C, Sun T, Huang S, He M, Liang B, Shen Z, et al. Personalized Machine Learning-Coupled Nanopillar Triboelectric Pulse Sensor for Cuffless Blood Pressure Continuous Monitoring. ACS nano. 2023;17(23):24242–58.

Yeh KY, Lin TH, Hsieh YY, Chang CM, Yang YJ, Lu SS, editors. A cuffless wearable system for real-time cutaneous pressure monitoring with cloud computing assistance2018.

Yen CT, Chang SN, Cai CY. Development of a Continuous Blood Pressure Measurement and Cardiovascular Multi-Indicator Platform for Asian Populations by Using a Back Propagation Neural Network and Dual Photoplethysmography Sensor Signal Acquisition Technology. 2021;2021.

Yen CT, Chang SN, Jia-Xian L, Huang YK. A deep learning-based continuous blood pressure measurement by dual photoplethysmography signals. 2022;70(2):2937–52.

Yi Z, Liu Z, Li W, Ruan T, Chen X, Liu J, et al. Piezoelectric Dynamics of Arterial Pulse for Wearable Continuous Blood Pressure Monitoring. Adv. 2022;34(16):e2110291.

Yi ZR, Zhang WM, Yang B. Piezoelectric approaches for wearable continuous blood pressure monitoring: a review. J Micromech Microeng. 2022;32(10):11.

Yoon YZ, Kang JM, Kwon Y, Park S, Noh S, Kim Y, et al. Cuff-Less Blood Pressure Estimation Using Pulse Waveform Analysis and Pulse Arrival Time. IEEE J Biomed Health Inform. 2018;22(4):1068–74.

Yousefian P, Shin S, Mousavi A, Kim CS, Mukkamala R, Jang DG, et al. The Potential of Wearable Limb Ballistocardiogram in Blood Pressure Monitoring via Pulse Transit Time. Sci. 2019;9(1):10666.

Yousefian P, Shin S, Mousavi AS, Tivay A, Kim CS, Mukkamala R, et al. Pulse Transit Time-Pulse Wave Analysis Fusion Based on Wearable Wrist Ballistocardiogram for Cuff-Less Blood Pressure Trend Tracking. IEEE Access. 2020;8:138077–87.

Zhang AH, Zhou XZ, Yang LM, Shen R, Wei Z. Continuous blood pressure measurement method based on the pulse image sensor and BP neural network. 2014. p. 476–9.

Zhang F, Yang K, Pei Z, Wu Y, Sang S, Zhang Q, et al. A highly accurate flexible sensor system for human blood pressure and heart rate monitoring based on graphene/sponge. RSC Adv. 2022;12(4):2391–8.

Zhang J, Wu D, Li Y. Cuff-less and Calibration-free Blood Pressure Estimation Using Convolutional Autoencoder with Unsupervised Feature Extraction. Annu Int Conf IEEE Eng Med Biol Soc. 2019;2019:3323–6.

Zhang K, Ling W. Health monitoring of human multiple physiological parameters based on wireless remote medical system. 2020;8:71146–59.

Zhang Q, Shen L, Liu P, Xia P, Li J, Feng H, et al. Highly sensitive resistance-type flexible pressure sensor for cuffless blood-pressure monitoring by using neural network techniques. 2021;226.

Zhang Y, Berthelot M, Lo B, editors. Wireless wearable photoplethysmography sensors for continuous blood pressure monitoring2016.

Zhang Y, Li J, editors. Performance analysis of big data transmission in wearable system based on special textile clothing2021.

Zhao T, Ye Z, Zhang T, Shi C, Mahdad AT, Wang Y, et al., editors. Continuous blood pressure monitoring using low-cost motion sensors on AR/VR headsets2022.

Zheng Y, Poon CC, Yan BP, Lau JY. Pulse Arrival Time Based Cuff-Less and 24-H Wearable Blood Pressure Monitoring and its Diagnostic Value in Hypertension. J Med Syst. 2016;40(9):195.

Zhou ZB, Cui TR, Li D, Jian JM, Li Z, Ji SR, et al. Wearable Continuous Blood Pressure Monitoring Devices Based on Pulse Wave Transit Time and Pulse Arrival Time: A Review. Materials (Basel). 2023;16(6):07.

Zhu Z, Li R, Pan T. Imperceptible Epidermal-Iontronic Interface for Wearable Sensing. Adv. 2018;30(6).

Zienkiewicz A, Vihriala E, Seppala E, Ferdinando H, Myllyla T, editors. Wearable sensor system on chest for continuous measurement of blood pressure and other vital signs2022.
